# P2X3 receptor antagonism attenuates the progression of heart failure

**DOI:** 10.1038/s41467-023-37077-9

**Published:** 2023-03-28

**Authors:** Renata M. Lataro, Davi J. A. Moraes, Fabio N. Gava, Ana C. M. Omoto, Carlos A. A. Silva, Fernanda Brognara, Lais Alflen, Vânia Brazão, Rafaela Pravato Colato, José Clóvis do Prado, Anthony P. Ford, Helio C. Salgado, Julian F. R. Paton

**Affiliations:** 1 Department of Physiological Sciences, Center of Biological Sciences, Federal University of Santa Catarina, Florianópolis, Santa Catarina Brazil; 2 Department of Physiology, Ribeirão Preto Medical School, University of São Paulo, Ribeirão Preto, Brazil; 3 Department of Clinical Veterinary, Agrarian Sciences Center, Londrina State University, Londrina, Brazil; 4 College of Pharmaceutical Sciences of Ribeirão Preto, University of São Paulo, Ribeirão Preto, Brazil; 5 CuraSen, 2655 Campus Dr #110, San Mateo, CA 94403 USA; 6 Manaaki Manawa—The Centre for Heart Research, Department of Physiology, Faculty of Medical & Health Sciences, University of Auckland, Auckland, New Zealand

**Keywords:** Target identification, Heart failure, Preclinical research

## Abstract

Despite advances in the treatment of heart failure, prognosis is poor, mortality high and there remains no cure. Heart failure is associated with reduced cardiac pump function, autonomic dysregulation, systemic inflammation and sleep-disordered breathing; these morbidities are exacerbated by peripheral chemoreceptor dysfunction. We reveal that in heart failure the carotid body generates spontaneous, episodic burst discharges coincident with the onset of disordered breathing in male rats. Purinergic (P2X3) receptors were upregulated two-fold in peripheral chemosensory afferents in heart failure, and when antagonized abolished these episodic discharges, normalized both peripheral chemoreceptor sensitivity and the breathing pattern, reinstated autonomic balance, improved cardiac function, and reduced both inflammation and biomarkers of cardiac failure. Aberrant ATP transmission in the carotid body triggers episodic discharges that via P2X3 receptors play a crucial role in the progression of heart failure and as such offer a distinct therapeutic angle to reverse multiple components of its pathogenesis.

## Abstract (web summary)

Despite medications, heart failure worsens with time with many patients dying within five years of diagnosis. Here the authors show that blocking purinergic receptors in the carotid body stops heart failure progression, improves its function, reduces sleep apneas and systemic inflammation in male rats.

## Introduction

Heart failure (HF) is a major public health problem and is one of the leading causes of death worldwide^1^. It is estimated that globally 38 million individuals have HF and its prevalence is expected to rise as life longevity increases^1,2^. HF involves a complex interaction of several mechanisms including neurohumoral and inflammation^3^ resulting in autonomic dysfunction^4–6^, cardiac and respiratory failure^7^ that all contribute to the morbidity and mortality of HF. The degree to which sympathetic activity is elevated in HF is strongly associated with its worsening prognosis^4–6^. Thus, understanding the origins of elevated sympatho-excitation in HF is critical, as is how to temper it, which remains unresolved.

Increased activity of peripheral chemoreceptors has been advanced as a mechanism for driving the excessive sympatho-excitation observed in HF and their sensitivity is strongly associated with mortality^7–11^. Additionally, sleep-disordered breathing and central apneas commonly found in patients with HF have also been ascribed to hyperreflexia (i.e. increased sensitivity) of the peripheral chemoreceptor reflex evoked responses^9–11^. Hence, carotid bodies have been considered as a potential new target to improve the quality of life and decrease mortality in HF patients. In rats with HF, bilateral ablation of the carotid bodies stabilized respiratory function, restored autonomic balance, including a significant reduction in sympathetic activity, reduced cardiac remodelling and improved ejection fraction, and survival^9^. In patients with HF, carotid body resection (uni-/ bi-lateral) also showed promising results, with a decrease in both sympathetic nerve activity and peripheral chemosensitivity, and an improvement in exercise tolerance and life quality^12^. Together, data from both experimental animals and humans reveal an important role for peripheral chemoreceptors in the pathogenesis of HF, and support the hypothesis that inhibition of their hyperactivity represents a novel therapeutic strategy.

Although surgical denervation of the carotid body in humans has provided important proof of concept data, such an approach may not be free from risks. In human HF, bilateral carotid body resection did not improve pump function and may have been associated with worsened sleep apnea resulting in greater falls in blood oxygen saturation over more prolonged periods^12^. This raises the question of whether there is a way to normalize the excitability of the carotid bodies without the need to remove them, and maintain their vital homeostatic functions. This would preserve the excitatory respiratory drive during periods of hypoxia/hypercapnia as experienced during sleep apnoea and provide autonomic modulation to support cardiac contractility, for example. The present study was designed to test such an approach.

Multiple transmitters are involved in carotid body signaling with adenosine tri-phosphate, ATP, being a major contributor^13,14^. In response to hypoxia, ATP is released from glomus cells within the carotid body providing increases in chemosensory afferent drive by acting on P2X receptors. These receptors are members of a family of ATP-gated ion channels consisting of seven receptor subunits: P2X1-P2X7^15^. One of these - the P2X3 receptor - has been associated with dysfunctional afferent signaling^16^. Nurse and colleagues were the first to describe the presence of P2X3-receptors in the carotid body and petrosal ganglion neurons in healthy rats^17^. Recently, these receptors were found to be elevated in neurons of the petrosal ganglion innervating the carotid body of hypertensive rats and following their inhibition both sympathetic activity and blood pressure were reduced^18^.

In the present study on rats with chronic heart failure, we tested the hypothesis that pharmacological antagonism of P2X3 receptors within the carotid body would normalize peripheral chemosensitivity, reduce sympathetic activity and reinstate respiratory stability thereby attenuating the progression of HF pathology.

## Results

### P2X3 receptors are upregulated in petrosal neurons mediating the chemoreflex in HF rats

**Fig. 1:**
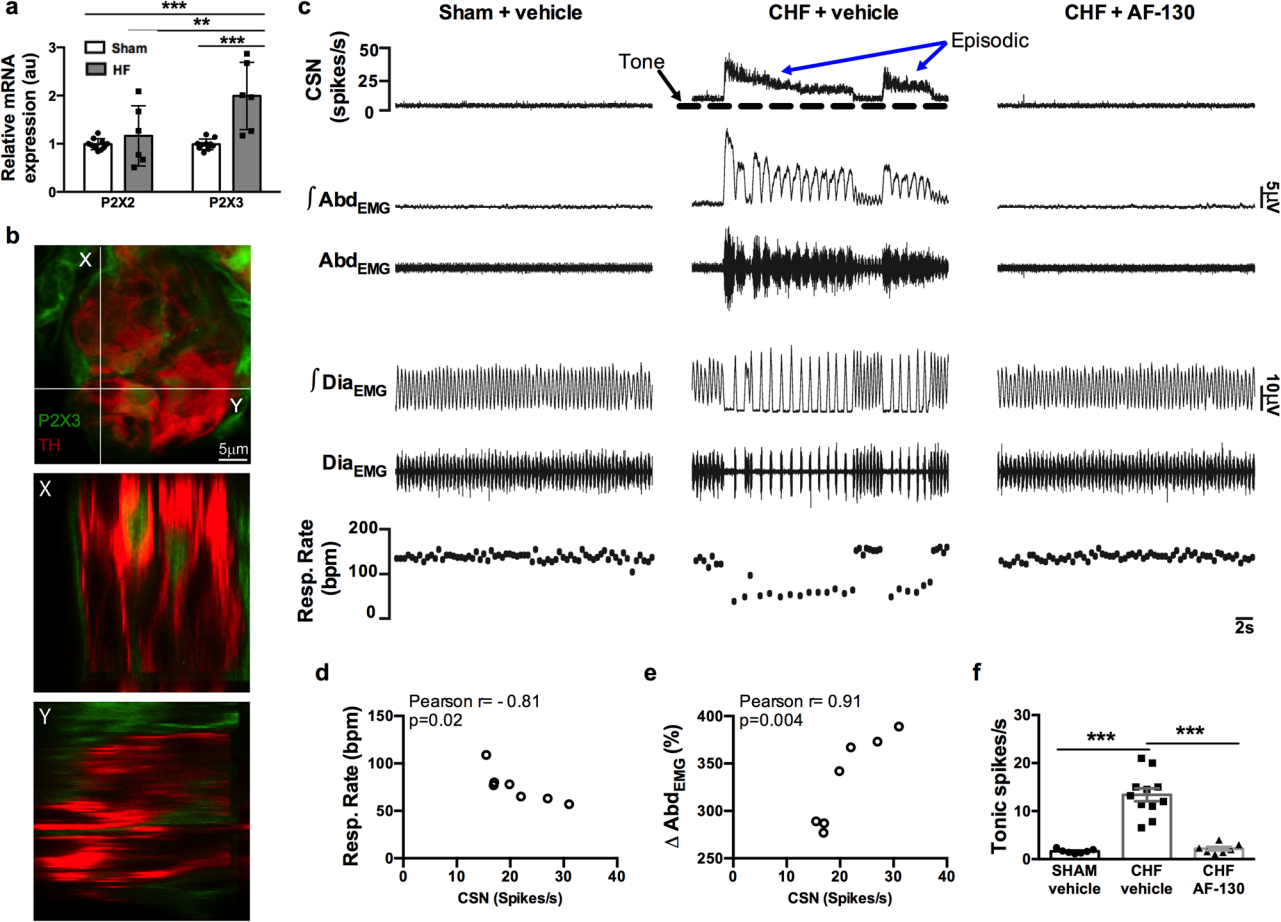
Episodic carotid sinus nerve discharge in chronic heart failure (CHF) rats is mediated by P2X3 receptors and causes respiratory disturbance via activation of active expiration. **a** RT–qPCR indicated upregulation of P2X3 but not P2X2 receptors in the petrosal ganglion chemoreceptive neurons from HF rats. β-actin was used as a house keeping control gene to normalize reactions. The relative quantitation was determined by the ΔΔCt method. Data shown as mean ± SD; *n* = 10 and 6 for sham and HF group, respectively. Two-way ANOVA Bonferroni post-test. **P < 0.01, ***P < 0.001. **b** Immunofluorescence of P2X3 receptors (green) and glomus cells expressing tyrosine hydroxylase (TH; red) within the carotid body of a heart failure rat. The top panel is a conventional image and two orthogonal views taken at positions X and Y are shown beneath. The absence of P2X3 receptor immunofluorescence superimposition with TH staining supports the viewpoint that P2X3 receptors are on sensory afferent fibres juxtaposed to the glomus cells. This was repeated in three rats. Scale bar 5 μm. For additional P2X3/TH immunofluorescence images see Fig S11. **c** Raw and integrated (∫) simultaneous recordings of carotid sinus nerve (CSN) activity, electromyographic (EMG) recordings from expiratory (Abd_EMG_) and inspiratory (Dia_EMG_) muscles in anaesthetized rats. Note the presence of episodic CSN discharge (blue arrows) coincident with both breathing irregularity and onset of active expiration in CHF rats. These changes were all prevented by AF-130 treatment (CHF + AF-130) as was the tonic CSN activity (arrowed). **d** and **e** show a correlation b**e**tween the level of CSN activity with respiratory rate (Resp. Rate, *n* = 7) and changes in activity of Abd_EMG_ (*n* = 7) over 10 min from chronic HF rats, respectively. **f** CSN tonic discharge is enhanced in chronic CHF rats and normalized by AF-130. Data shown are mean ± SD; *n* = 7 for sham vehicle; *n* = 11 for CHF vehicle a*n*d *n* = 7 for CHF AF-130 group. Data were tested for normality (Kolmogorov–Smirnov test) and compared using One-way ANOVA Bonferroni post-test. ***P < 0.001. Correlations were assessed using Pearson’s correlation coefficients, two-sided. Source data are provided as a Source Data file.

Juvenile rats were studied ten days after ligating the left anterior descending coronary artery, which resulted in a 39 ± 4.8% (*n* = 17, Fig S1a, b) infarcted area localized to the left ventricle, as revealed by *post hoc* histological analysis (Fig S1a), and a reduced ejection fraction compared to sham animals (25 ± 9 *vs* 68 ± 10%, *p* = 0.001, *n* = 9). We analyzed the expression of mRNA of P2X3 and P2X2 receptors from physiologically identified petrosal ‘chemoreceptive’ neurons (identified by their response to potassium cyanide) from HF (*n* = 6 neurons) and sham rats (*n* = 10 neurons) using RT–qPCR. We found an approximately two-fold increase in expression of P2X3 receptors chemoreceptive petrosal neurons of HF *versus* sham rats (Table S1; Fig. 1a, *P* < 0.0003); P2X2 receptor expression was similar between both groups (Fig. 1a). In contrast, in HF and sham rats, petrosal neurons not responding to carotid body stimulation had similar levels of P2X3 receptor mRNA expression (Fig S1e). Resting membrane potential of petrosal chemoreceptor neurons were more depolarized in HF (−46 ± 3.6 mV) than both sham animals (−57 ± 3 mV) and non-chemoreceptor neurons (Table S1; Fig S1f). Moreover, immunofluorescence to detect P2X3 receptor expression was abundant in the carotid bodies of HF rats (Fig. 1b & S11). The functional significance of the upregulated P2X3 receptor mRNA in HF rats was tested physiologically as detailed below.

### Carotid body afferent recordings reveal spontaneous, episodic discharges in chronic HF rats

AF-130, a highly selective P2X3 receptor antagonist with minimal brain penetrance, was administered (30 mg/kg/day, s.c.) three days after myocardial infarction for 7 weeks in chronic HF rats; data were compared to chronic HF and sham-operated controls both given vehicle. Chronic HF was induced by ligation of the left anterior descending coronary artery and infarct size measured 8 weeks later was not different between vehicle versus drug-treated groups (42 ± 8 *versus* 45 ± 8%, respectively; n.s., Fig S1a, b). After eight days of administration, PK of AF-130 was assessed. The plasma concentration of AF-130 was analyzed at 1, 4, and 24 h post-administration (Fig S1c, *n* = 6): one hour after administration, plasma AF-130 concentration was 4.187 ± 569 ng/mL, which dropped by 50.4% four hours later (Fig S1c). There was no difference in actual weight between animal groups (drug-treated: 518 ± 67, vehicle: 548 ± 47 g, n.s.; Fig S1d).

Carotid sinus nerve activity, as an index of carotid body excitability, was recorded simultaneously with electromyographic activity from both the diaphragm (Dia_EMG_, inspiratory) and abdominal muscles (Abd_EMG_, active expiratory; Fig. 1b) in anaesthetized rats breathing spontaneously either with (*n* = 4) or without trachea cannulation (*n* = 7) eight weeks after myocardial infarction. Recordings of the carotid sinus nerve exhibited unexpected, episodic burst discharges that occurred spontaneously at a frequency of 17.9 ± 5.3 events/10 min and of variable duration (6–23 s; 13.9 ± 6.4 s) and persisted for the duration of the recording (>1.5 h; Fig. 1b). These episodic discharges correlated with the onset of respiratory de-stabilization/hypopnea as revealed by the variable duration and reduced burst frequency of Dia_EMG_ activity (Fig. 1b, c; *r* = −0.81; *p* = 0.02) and with the appearance of burst discharges in Abd_EMG_ activity (*r* = 0.91; P = 0.004, Fig. 1b, d; *n* = 7), and caused increases in heart rate (398 ± 28 to 448 ± 24.8 bpm, *P* < 0.05); the latter indicative of cardiac sympathetic activation. Additionally, tonic levels of carotid sinus nerve activity were greater in rats with chronic HF (*n* = 11) relative to sham (n = 7) controls (13.4 ± 4.4 *vs* 1.64 ± 0.4 spikes/s, Fig. 1a, e; *p* < 0.0001). In all chronic HF rats, systemic P2X3 receptor antagonism (*n* = 7) abolished the episodic carotid sinus nerve bursting (Fig. 1b) and reduced its tonic activity to levels comparable to those in sham controls (2.18 ± 1.0 spikes/s, Fig. 1b, e). In the absence of episodic discharges, both the duration and frequency of Dia_EMG_ stabilized completely and Abd_EMG_ bursting was abolished. Given that cardiac arrhythmia were at least twenty times less infrequent (2.33 ± 1.75 arrhythmias per 30 min) than the occurrence of aberrant carotid sinus nerve discharges (17.9 ± 5.3 events/10 min) these novel data suggest that in chronic HF *both* spontaneous episodic *and* tonic activity is generated by the carotid body and caused by ATP acting on P2X3 receptors rather than a consequence of reduced blood flow to carotid body triggered by the arrhythmia. We surmised that these episodic discharges trigger inspiratory destabilization through activation of pathways promoting aberrant expiratory activity. Since this supposition was based on data from anesthetized rats, we tested its plausibility in conscious chronic heart failure animals.

### Systemic P2X3-receptor antagonism reinstates respiratory stability in conscious chronic HF rats

**Fig. 2:**
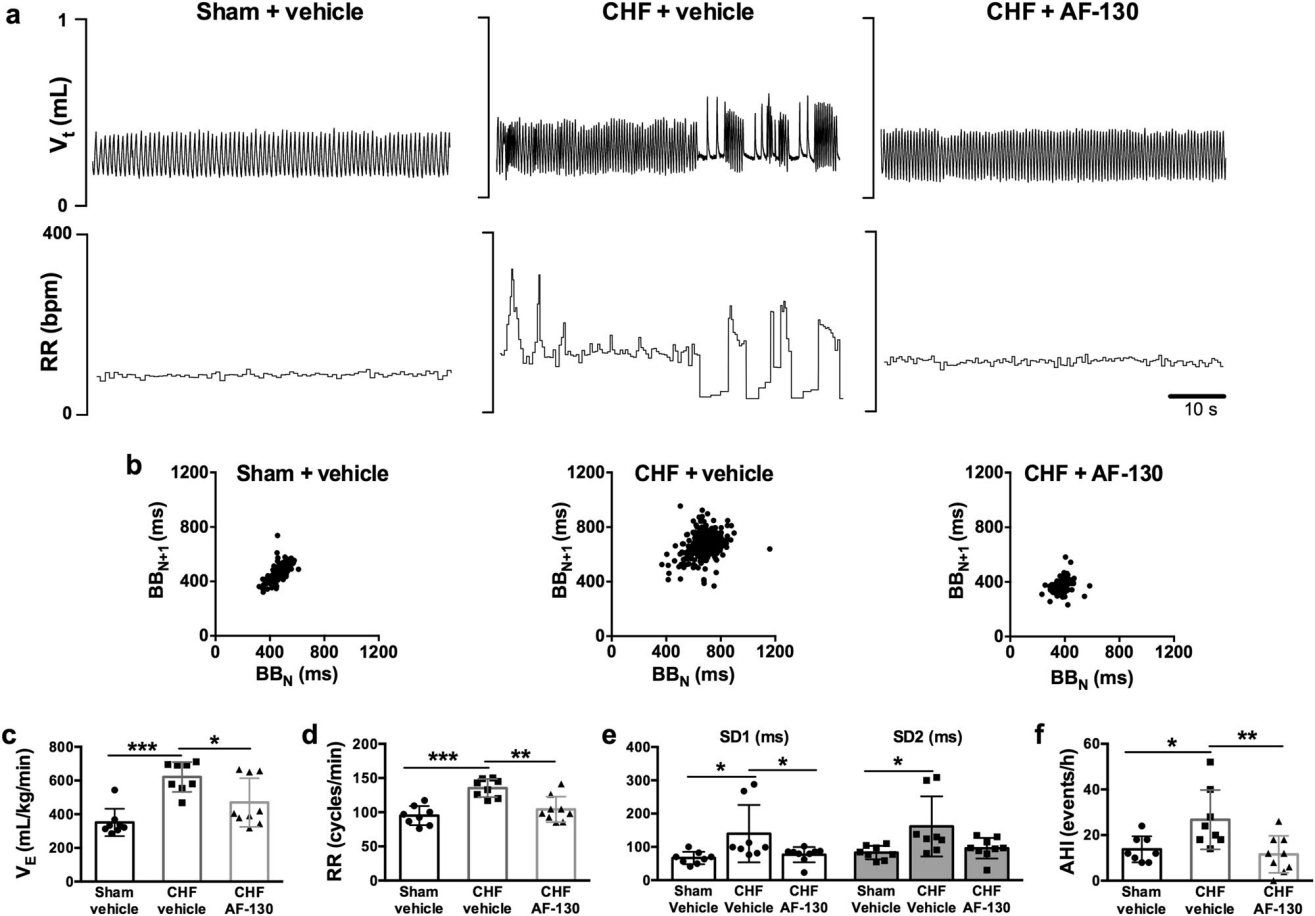
Chronic P2X3-receptor antagonism restored normal breathing pattern in chronic heart failure (CHF) rats. **a** Representative tracings displaying tidal volume (V_t_) and respiratory frequency (RR) recorded in conscious rats using plethysmography and **b** Poincaré plots for breath-to-breath interval (BB_N_) versus the subsequent interval (BB_N+1_). Breathing instability in CHF rats is demonstrated in V_t_ and RR tracings, and higher breathing variability in CHF rats. P2X3-receptor antagonism restored normal breathing rhythm in HF rats (**a**, **b**) and reduced minute ventilation (V_E_), respiratory frequency (RR), short- and long-term breathing interval variability (SD1 and SD2), and the incidence of apnoea and hypopnoea (AHI) in CHF rats (**c–f**). Data are shown as mean ± SD. One-way ANOVA Tukey post-test; *n* = 8 for sham vehicle, *n* = 8 for CHF vehicle, and *n* = 9 for CHF AF-130 group. *P < 0.05, **P < 0.01, ***P < 0.001. Source data are provided as a Source Data file.

Breathing was measured using plethysmography and was unstable in chronic HF rats treated with vehicle (Fig. 2a) as reflected in the Poincare plots (Fig. 2b). These respiratory irregularities produced increases in the PaCO_2_ (42.4 ± 1.2 vs 38.5 ± 0.7 mmHg; *p* < 0.0001, Fig S2a) and reductions in PaO_2_ (74.6 ± 5.9 vs 87.7 ± 2.1 mmHg; *p* < 0.0001, Fig S2b) in chronic HF (*n* = 6) relative to sham controls (*n* = 5); no difference in blood gases were seen in HF animals during normal breathing relative to sham controls (Fig S2a, b). Thus, the hypercapnia and hypoxia was a consequence, not a cause, of the breathing disturbance. When compared to sham controls (*n* = 8), chronic HF rats (*n* = 8) showed higher: minute ventilation (621 ± 89 *vs* 351 ± 81 mL/kg/min, *p* < 0.001, Fig. 2c), respiratory frequency (135 ± 13 *vs* 95 ± 14 cycles/min, *p* < 0.001, Fig. 2d), short- and long-term breathing interval variability (SD1, 140 ± 86 *vs* 67 ± 19 ms, *p* < 0.05; SD2, 162 ± 90 *vs* 83 ± 20 ms, *p* < 0.05, Fig. 2e) and incidence of apneas-hypopneas (27 ± 13 *vs* 14 ± 6 events/h, *p* < 0.05, Fig. 2f); these characteristics are all consistent with hyperexcitability of the carotid body. After AF-130 treatment in chronic HF rats (*n* = 9) there were marked reductions in: minute ventilation (470 ± 144 mL/kg/min, *p* < 0.05), respiratory frequency (104 ± 19 cycles/min, *p* < 0.01), SD1 (77 ± 23 ms, *p* < 0.05), and episodes of apneas-hypopneas (12 ± 8 events/h, *p* < 0.01, Fig. 2a–f); these values approached those in the sham group (Fig. 2a–f). Further examination indicated apnea rates were reduced by AF-130 (7.9 ± 5.8 vs vehicle CHF 19.6 ± 15.3 events/h, *p* < 0.05, Fig S3a) but not their duration (Fig S3b); also there was no change in hypopneas when analyzed separately (Fig S3c). AF-130 changed neither respiratory tidal volume (Fig S3e) nor sigh frequency (Fig S3d). All told, the data indicate blockade of P2X3 receptors rescues much of the pathological breathing in chronic HF. We next asked whether antagonizing these receptors within the carotid body would also normalize the autonomic and respiratory imbalance found in HF.

### Reversal of autonomic dysfunction after blockade of P2X3 receptors in the carotid body of HF rats in situ

**Fig. 3:**
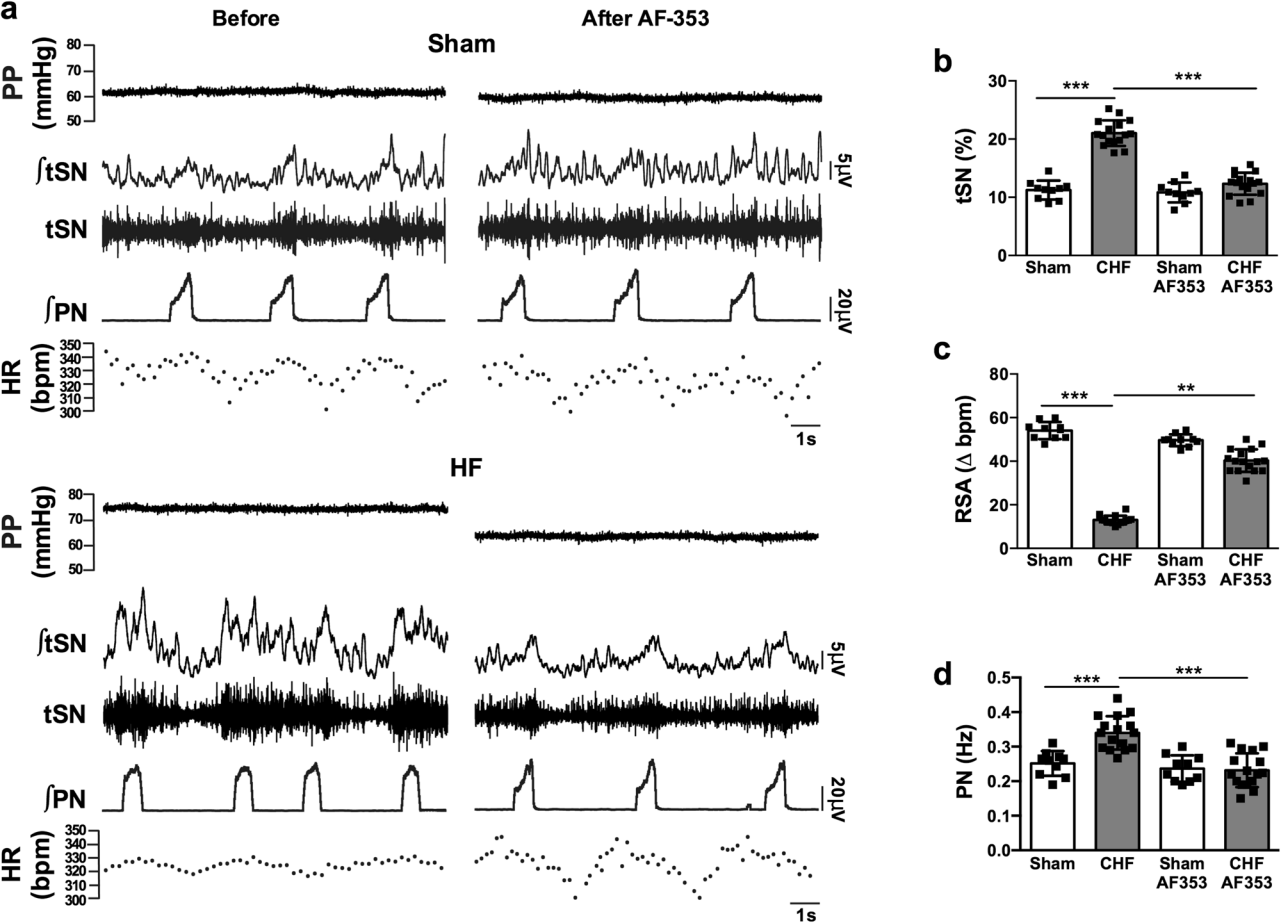
P2X3-receptor antagonism in heart failure (HF) rats is associated with restoring autonomic balance and respiratory activity in the in situ preparation. **a** Raw (∫) and integrated records of thoracic sympathetic (tSN), phrenic nerve (PN), and heart rate (HR, bpm) in sham and HF rats before and after P2X3 receptor inhibition using AF-353. Note the drug was infused via a micropipette into the carotid bodies directly. In HF rats, there was increased activity of tSN and PN, associated with a reduced magnitude of respiratory sinus arrhythmia (RSA) compared to shams. Average values of **b** tSN, **c** respiratory sinus arrhythmia (RSA), and **d** PN frequency from sham and HF rats before and after AF-353 delivery. Data are shown as mean ± SD; *n* = 10 and 16 for sham and HF group, respectively. One-way ANOVA Bonferroni post-test. **P < 0.01, ***P < 0.001. Source data are provided as a Source Data file.

We first performed whole cell recordings of physiologically characterized chemoreceptive primary afferent petrosal ganglion neurons in arterially perfused in situ rat preparations ten days after ligating the left anterior descending coronary artery (HF) or the sham surgery. The chemoreceptive petrosal neurons from HF (*n* = 6 cells) versus sham animals (*n* = 10 cells) were more depolarized (−46.3 ± 3.6 *vs* −56.6 ± 6 mV; *p* < 0.001, Fig S4a, b) and exhibited tonic firing (2.2 ± 1.3 *vs* 0 Hz; *p* < 0.001, Fig S4a, d). Next, assessments were made on baseline autonomic and respiratory variables after focal delivery of a highly selective, non-competitive P2X3-receptor antagonist (AF-353; 15 nl, 20 mM) infused via a glass pipette placed into the carotid bodies bilaterally. This resulted in: (i) a reduction of thoracic sympathetic nerve activity (tSN; from 21 ± 2.2 to 12.3 ± 1.9%, Fig. 3a, b, *p* < 0.001) in HF (*n* = 16) to levels not different to those in sham (*n* = 10) rats (before 11.2 ± 1.6 vs after drug 10.8 ± 1.7%, Fig. 3a, b; n.s.); (ii) a reduction of perfusion pressure (PP; from 75.3 ± 3.3 to 64 ± 4 mmHg; *p* < 0.0001) to levels not different to those in sham rats (before 62.3 ± 2.9 vs after drug 60 ± 5.5 mmHg; n.s.; Fig. 3a); (iii) augmentation of the amplitude of respiratory sinus arrhythmia (from 13 ± 2 to 40 ± 5 bpm, Fig. 3a, c; *p* < 0.01), which approached the level of sham animals (54 ± 4 bpm, n.s.) and revealed an improvement in heart rate variability/increased vagal tone; (iv) a lowering of phrenic nerve (PN) frequency from 0.34 ± 0.05 to 0.23 ± 0.05 Hz (Fig. 3a, d**;** *n* = 10, *p* < 0.001), -a level similar to that found in sham rats (0.25 ± 0.04 Hz; Fig. 3a, d; *n* = 10, n.s.). In contrast to HF rats, no effect of the antagonist was observed on tSN, respiratory sinus arrhythmia or PN frequency in sham rats (Fig. 3a–d). Vehicle infusions into the carotid body were without effect on both basal levels and chemoreflex evoked tSN and PN activities (Fig S5a–e). Thus, P2X3 receptors within the carotid body generate aberrant tonic afferent drive causing autonomic and respiratory imbalances in HF. Whether these receptors also contribute to carotid body hypersensitivity (autonomic and/or respiratory responses) was addressed next.

### Carotid body afferent hyperresponsiveness in rats with chronic HF is mediated by P2X3 receptors

**Fig. 4:**
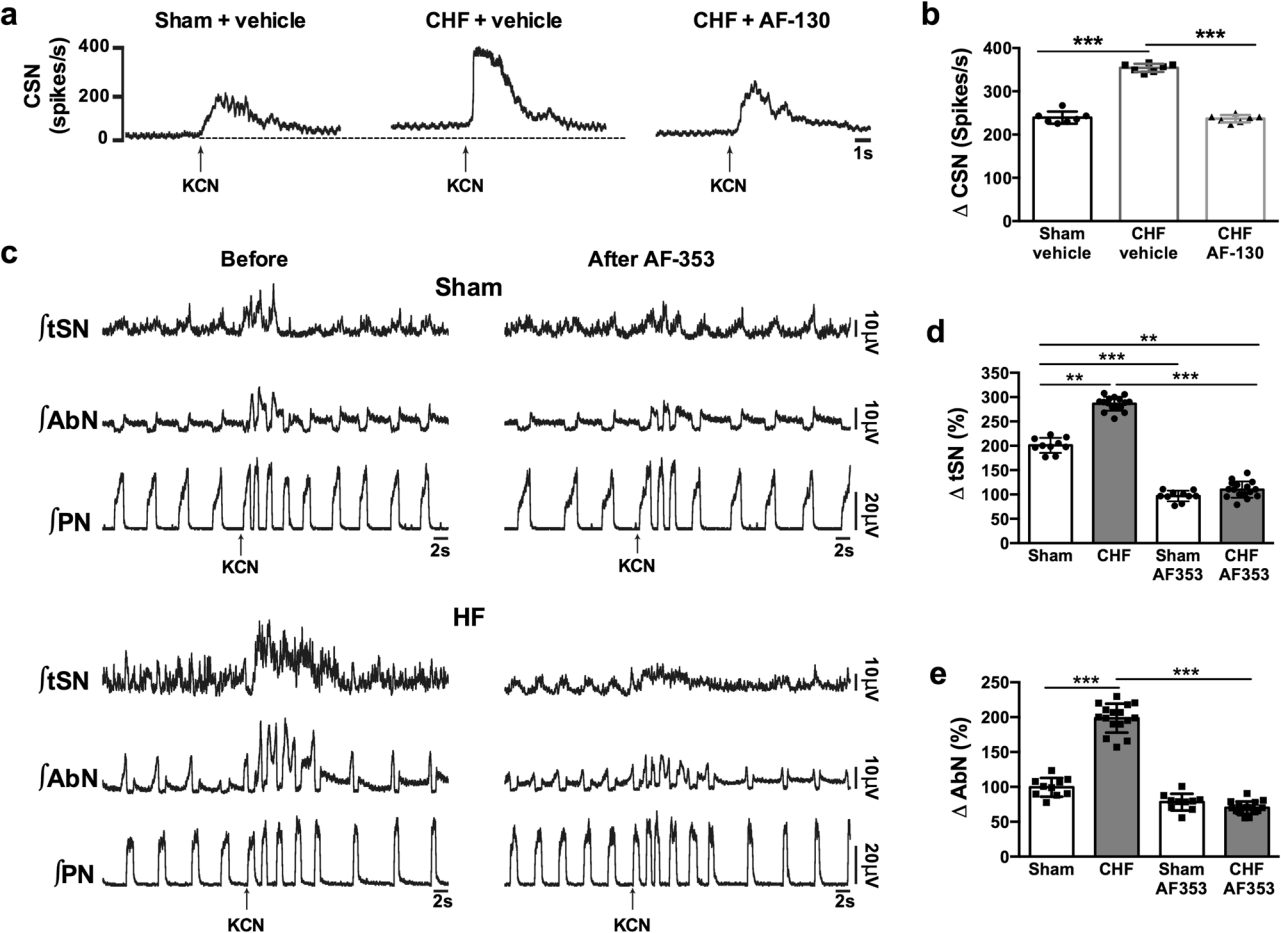
Carotid body P2X3-receptors mediate afferent hypersensitivity and chemoreflex hyperreflexia in heart failure (HF) rats. **a** Integrated (∫) recordings of carotid sinus nerve (CSN) activity during activation of peripheral chemoreceptors (potassium cyanide - KCN; 0.05 ml, i.v., 0.05%) in anaesthetized rats. Note presence of elevated tonicity (relative to sham and dotted line) and augmented evoked response in CSN **b** in the chronic HF (CHF) rats and that these were both normalized after P2X3 receptor blockade (**a**, **b**; drug was given systemically). Data are shown as mean ± SD and were tested for normality (Kolmogorov–Smirnov test) and compared using One-way ANOVA Bonferroni post-test. *n* = 7 per group; ***P < 0.001. The chemoreflex hyperreflexia of both the thoracic sympathetic (tSN) (**c**, **d**), and abdominal nerve (AbN) activity (**c**. **e**), was normalized by P2X3 receptor blockade in the carotid bodies performed by microperfusion of antagonist in the in situ preparation. ∫PN integrated phrenic nerve activity. Data are shown as mean ± SD; *n* = 10 and 16 for sham and HF group, respectively. One-way ANOVA Bonferroni post-test. **P < 0.01, ***P < 0.001. Source data are provided as a Source Data file.

In anesthetized chronic HF (*n* = 7) rats (8 weeks since myocardial infarction), the carotid bodies were stimulated with low dose potassium cyanide boluses (i.v.) and displayed greater increases in evoked carotid sinus nerve discharge than sham (*n* = 7) controls (354 ± 9.4 *vs* 239 ± 14 spikes/s; Fig. 4a, b; *p* < 0.0001). Following systemic P2X3 receptor antagonism over 7 weeks, the carotid body evoked afferent volley in chronic HF (*n* = 7) rats was reduced to levels seen in sham controls (237 ± 8.5 spikes/s; Fig. 4a, b). These data suggest that in chronic HF rats carotid body afferent hyperresponsiveness is, in large part, mediated by ATP acting on P2X3-receptors. We next determined whether antagonizing these receptors after focal delivery of P2X3-receptor antagonist into the carotid bodies reduced the chemoreflex evoked sympathetic and respiratory responses.

### Hyperreflexia of chemoreflex evoked responses is normalized by antagonism of P2X3 receptors in the carotid body

Ten days post-myocardial infarction, we used the in situ preparation and assessed the carotid body evoked responses of chemoreceptive petrosal neurons in HF and sham rats, as well as the chemoreflex evoked sympathetic and respiratory motor responses before/after bilateral infusion of a P2X3 receptor antagonist infusion directly into the carotid bodies. The chemoreceptive petrosal neurons from HF rats displayed an enhanced firing response compared to sham (55.1 ± 8.8 vs 22.3 ± 4.7 spikes; *p* < 0.0001; Fig S4a, c). Chemoreflex evoked increases in tSN were 286.1 ± 14.3% and 200.7 ± 15.3% for HF (*n* = 16) and sham (*n* = 10) rats, respectively (*p* < 0.0001), and after P2X3 receptor blockade were reduced to 109.9 ± 16.6% and 96.7 ± 10.9% in HF and sham rats, respectively (Fig. 4c, d, *p* < 0.0001); these new values were not different to one another. Most tSN was positively modulated in the expiratory phase (summed post-inspiration and late expiration) and this was reduced from 219 ± 20 to 79 ± 12% in HF rats (Fig. 4c, *p* < 0.01) and from 128 ± 6 to 54 ± 12% in sham controls (Fig. 4c, *p* < 0.05). Overall, the reduction in expiratory modulated tSN was greatest in the HF group (*p* < 0.001). The chemoreflex evoked increases in post-inspiratory and late expiratory activities recorded from the AbN were summed and greater in the HF versus sham rats (198.5 ± 20 *vs* 99.3 ± 13%, Fig. 4c, e; *p* < 0.001). P2X3 receptor blockade reduced the AbN chemoreflex evoked expiratory discharge to 70.1 ± 8.9% (Fig. 4c, e; *p* < 0.001 compared to HF), a value not different to that observed in sham rats post drug (78.13 ± 11.9%, *p* > 0.05). On the other hand, the chemoreflex evoked PN discharge were not different between HF and sham animals before (0.24 ± 0.06 *vs* 0.24 ± 0.07 Hz; HF vs sham) or after P2X3 receptor blockade (0.24 ± 0.09 *vs* 0.25 ± 0.06 Hz; HF vs sham; Fig. 4c). These data provide support that in HF carotid body P2X3 receptors are mostly responsible for the hyperreflexia of sympathetic and expiratory chemoreflex responses in HF rats. Given these positive outcomes from the in situ rat, it was important to determine whether such responses translated to conscious rats.

### P2X3-receptor antagonism improves cardiac autonomic balance in conscious chronic HF rats

**Fig. 5:**
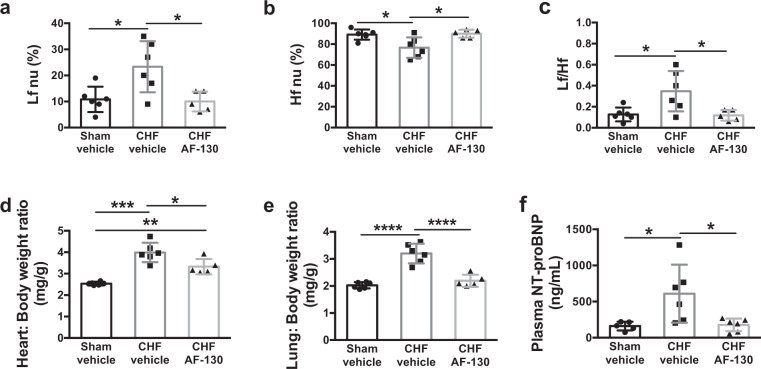
Chronic treatment with AF-130 improved heart rate variability (HRV) and reduced heart failure (HF) severity in chronic HF (CHF) rats. HRV was examined in the frequency domain (Hz) by spectral analysis. AF-130 treatment improved the cardiac sympathovagal modulation in CHF rats. **a** Lf: Low-frequency power; **b** Hf: high-frequency power and **c** Lf/Hf; nu: normalized units. P2X3-receptor antagonism attenuated the increase in **d** heart weights/body weight ratio, and prevented the rise in **e** lung /body weight ratio and **f** plasma N-Terminal Pro-B-Type natriuretic peptide (NT-proBNP), indicating that the treatment with AF-130 attenuated the HF progression in these animals. Data are shown as mean ± SD. One-way ANOVA Tukey post-test; (**a**–**e**) *n* = 6 for sham vehicle, *n* = 6 for CHF vehicle and *n* = 5 for CHF AF-130 group; (**f**) *n* = 5 for sham vehicle, *n* = 6 for CHF vehicle and *n* = 6 for CHF AF-130 group. *P < 0.05, **P < 0.01, ***P < 0.001. Source data are provided as a Source Data file.

Since P2X3 receptor blockade in the carotid body of in situ rats with HF improved respiratory sinus arrhythmia (see Fig. 3c), we assessed heart rate variability (HRV) using spectral analysis in conscious chronic HF rats. Our first analysis normalized the data using percentage change: in chronic HF rats (8 weeks post-myocardial infarction; *n* = 6), compared to sham animals (*n* = 6) there was: (i) an increased low-frequency power (23 ± 10 *vs*. 11 ± 5%, *p* < 0.05, Fig. 5a); (ii) a decreased high-frequency power (77 ± 10 vs. 89 ± 5%, *p* < 0.05, Fig. 5b) and (iii) a higher low/higher power ratio (0.35 ± 0.2 vs*.* 0.13 ± 0.07%, *p* < 0.05, Fig. 5c) in heart rate. As expected, these data support dominance of cardiac sympathetic over parasympathetic tone in chronic HF rats. Following systemic P2X3 receptor blockade in chronic HF rats, all HRV values were normalized to comparable levels seen in sham rats (Fig. 5a–c). In contrast, although un-normalized raw data showed similar trends no significant differences were seen following drug administration (Fig S6). We next sought to determine whether there was an improvement in cardiac pump function.

### Chronic systemic blockade of P2X3 receptors attenuates HF progression

**Fig. 6:**
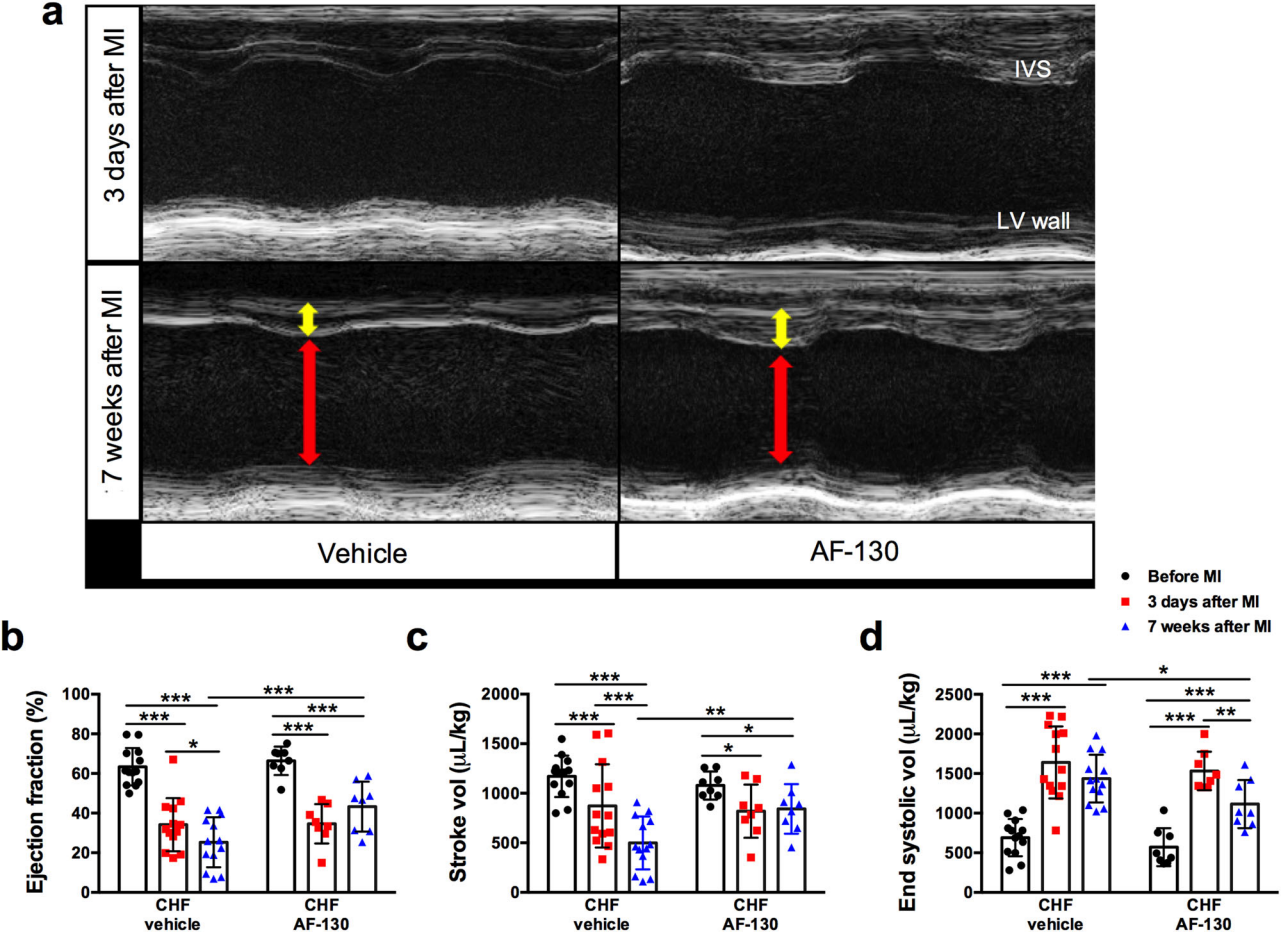
P2X3-receptor antagonism improves cardiac function in chronic heart failure (CHF) rats. **a** Representative images of echocardiography in rats submitted to myocardial infarction (MI), before and after 7 weeks of treatment with vehicle or AF-130. Red arrows indicate diastolic ventricular diameter and yellow arrows indicate diastolic ventricular wall thickness. P2X3-receptor antagonism prevented the reduction of ejection fraction (**b**) and stroke volume (**c**) during HF development, and reduced left ventricular (LV) end-systolic volume (**d**). The parameters were analyzed before MI surgery, three days after MI and after 7 weeks of vehicle (*n* = 13) or AF-130 (*n* = 8) administration. IVS: interventricular septum. Data are mean ± SD. Repeated measures two-way ANOVA, with Student-Newman-Keuls post hoc comparison. *P < 0.05, **P < 0.01, ***P < 0.001. Source data are provided as a Source Data file.

Compared to vehicle-treated rats, chronic (7 weeks, *n* = 8), administration of AF-130 to HF animals revealed increases in both ejection fraction (43 ± 13 vs*.* 25 ± 13%, Fig. 6a, b, *p* < 0.001) and stroke volume (843 ± 250 *vs*. 499 ± 267 μL/kg, Fig. 6c, *p* = 0.01), which was associated with a reduced end-systolic volume (1115 ± 304 vs. 1436 ± 300 μL/kg, Fig. 6d, *p* = 0.027). Additionally, drug-treated chronic HF rats had reduced cardiac hypertrophy as indicated by their lower heart/body weight ratio compared to vehicle control rats (3.3 ± 0.36 vs. 4 ± 0.45 mg/g respectively, Fig. 5d, *p* < 0.05) and lung/body weight ratio (2.2 ± 0.2 vs. 3.2 ± 0.37 mg/g, respectively, Fig. 5e, *p* < 0.0001); the latter indicative of reduced pulmonary edema. Drug treated chronic HF rats also showed a pronounced reduction in plasma levels of N-terminal pro-B-type natriuretic peptide (177 ± 88 vs*.* vehicle controls 609 ± 402 ng/g, Fig. 5f, *p* < 0.05). Thus, P2X3 receptor antagonism prevents the deleterious progression of cardiac dysfunction after myocardial infarction. Mechanistically this may be associated with reduced systemic inflammation.

### Chronic blockade of P2X3 receptors attenuates systemic inflammation and cytokines in chronic HF

**Fig. 7:**
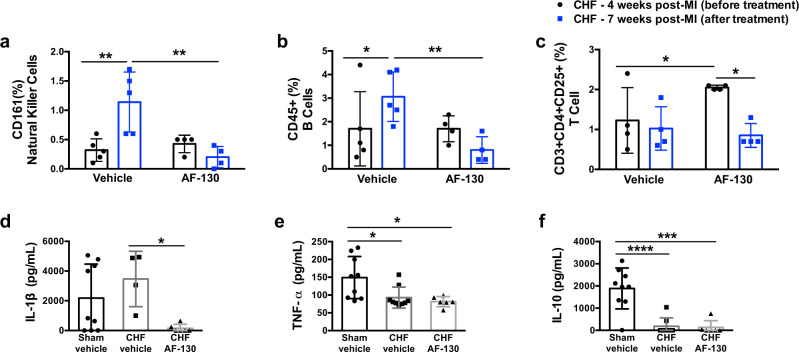
P2X3 receptor blockade reduces the immune cell response and plasma cytokine levels in chronic heart failure (CHF) rats. Quantitative data for natural killer cells (CD161, **a**), B cells (CD45+, **b**) and T cells (CD3 + CD4 + CD25+, **c**). These specific immune cell types were suppressed by antagonizing P2X3 receptors systemically in CHF rats. The immune cells were analyzed after three weeks of vehicle (*n* = 5) or AF-130 (*n* = 4) administration. Two-way repeated measures ANOVA, with Tukey post hoc comparison. Plasma levels of IL-1β (**d**), TNF-α (**e**), and IL-10 (**f**) in sham vehicle (*n* = 9) and CHF rats treated with either vehicle (*n* = 4, 9, and 8, respectively) or AF-130 (*n* = 6) are shown; administrations lasted for seven weeks. One-way ANOVA, with Tukey post hoc comparison. Data are mean ± SD. *P < 0.05, **P < 0.01, ***P < 0.001, ****P < 0.001. Source data are provided as a Source Data file.

AF-130 (2 mg/kg/h, iv, *n* = 4) or vehicle (*n* = 5) administration started four weeks after myocardial infarction and continued for three weeks; blood samples were taken just before the drug treatment and again after three weeks on drug (Fig. 7a–c). In vehicle-treated animals, there was an increase in natural killer cells (0.32 ± 0.2 vs. 1.14 ± 0.5%, *n* = 5, *p* < 0.01), B cells (1.7 ± 1.6 *vs* 3.1 ± 1%, *n* = 5, *p* < 0.05) but no change in T cells (1.22 ± 0.82 vs*.* 1.02 ± 0.54%, *n* = 4, *p* > 0.05; Fig. 7a–c) with the progression of HF (i.e. from four to seven weeks). This increase was prevented by AF-130 (4 vs. 7 weeks post-myocardial infarction: natural killer cells: 0.42 ± 0.15 *vs* 0.2 ± 0.2%, *n* = 4; B cells: 1.7 ± 0.5 vs. 0.8 ± 0.6%, *n* = 4; T cells: 2.05 ± 0.06 vs. 0.85 ± 0.3%, *n* = 4; all *p* < 0.05, Fig. 7a–c). Plasma cytokines were analyzed in separate sham and HF rats treated with either vehicle or AF-130 (30 mg/kg/day, s.c.) for 7 weeks. IL-1β was reduced with AF-130 treatment (149 ± 265 *vs* 3466 ± 1863 pg/mL, *n* = 4–6, *p* < 0.05; Fig. 7d), however, TNF-α (81 ± 14 vs. vehicle 93 ± 30, *n* = 6–9) and IL-10 (132 ± 304 vs. vehicle 183 ± 377, *n* = 6–8) were not changed (Fig. 7e, f). Additional analysis was made in HF rats receiving either vehicle or drug for seven weeks; the following immune cells were either depressed: CD3 + CD4 + CD8- (4.1 ± 1.6 *vs* 1.15 ± 1%, *p* = 0.01), CD4-CD8 + (1.34 ± 0.5 *vs* 0.55 ± 0.4%, *p* < 0.05) and CD8 + CD28 + (0.76 ± 0.3 vs. 0.05 ± 0.06%, p = 0.006; Fig S7a–c) or unaffected by drug treatment (e.g. CD4 + CD11a+; CD8 + CD11a+; CD4+CD28+; Fig S7d–f). These data indicate that specific immune cell types and IL-1β are suppressed by antagonizing P2X3 receptors in chronic HF rats. However, despite a reduced immune response, myocardial fibrosis was similar between AF-130 and vehicle treated chronic HF rats (0.42 ± 0.08 vs. 0.45 ± 0.08, *n* = 6 and 4, respectively, Fig S8).

## Discussion

In 1959, Holton^19^ first proposed that ATP was released by sensory nerves and eleven years later Burnstock described it as a transmitter in autonomic nerves^20^. Thirty years on, ATP was reported as a major transmitter substance released by the carotid body to activate sensory endings of the sinus nerve afferent fibers^21^. Subsequently, P2X2 and P2X3 subunits were identified in carotid body afferent terminals and petrosal somas^17^. P2X receptors containing the P2X2 receptor subunit were found to play an essential role in the physiological ventilatory responses to hypoxia in healthy mice^22^ whereas P2X3-subunit-containing receptors were the basis for pathological sensitization of carotid body reflexes^18^ as has been found in many other sensory systems^16^. For the first time in HF rats, the present study finds increased expression of P2X3 receptors on carotid body petrosal neurons that contribute to both its aberrant tonicity and hypersensitivity of reflex responses following stimulation.

A major co-morbidity of HF is respiratory instability with hypopneas and apneas^23,24^ which are associated with morbidity, mortality and reduced quality of life^25–27^. This breathing instability is cyclical and associated with increased carotid body chemoreflex sensitivity and occurs in at least 60% of HF patients^28,29^. This cyclical pattern of hypopnea/apnea is thought to be due to the hypersensitization of the peripheral chemoreceptors such that a period of hyperventilation-induced hypocapnia is followed by a period of disturbed breathing resulting from the reduced chemical drive from carbon dioxide (e.g. Cheyne Stokes breathing). In the present study, chronic HF rats presented hypopneas and apneas with increased expiratory motor activity causing substantive disruption to normal breathing (Figs. 1, 2). An unexpected and new insight was that the carotid bodies emit spontaneous, episodic discharges in HF rats. These are unlikely to be due to cyclical periods of hyperventilation/hypocapnia as recordings from the carotid sinus nerve revealed that this aberrant episodic discharge always preceded the onset of hypopneas and apneas. Moreover, these occurred when the trachea was intubated suggesting that the respiratory disturbance was not caused by upper airway collapse. Further, our blood gas analysis suggests that the hypoxia/hypercapnia resulted from respiratory disturbances, rather than triggering them. The episodic discharges are unlikely to be caused by cardiac arrhythmias triggering reduced carotid body blood flow as the frequency of occurrence of arrhythmias was twenty times slower that of the carotid sinus nerve bursts. As the episodic carotid sinus nerve discharge and respiratory instability were both blocked by focal P2X3 receptor antagonism within the carotid body (Fig. 1), we propose that the episodic discharge is caused by an intervallic release of ATP from glomus cells; this may be triggered by the sympathetic nervous system innervation of the carotid body.

Of further interest was the emergence of ‘active’ expiratory abdominal motor activity coincident with episodic carotid sinus nerve activity (Fig. 1). We suggest that the episodic discharge from the carotid body drives selectively reflex pathways activating expiration preferentially (not inspiration) triggering hypopneas/apneas in HF. This respiratory disturbance was abolished by P2X3 receptor blockade (Fig. 4) but it remains unclear why ATP is released episodically from the carotid body in HF unless they are under perfused and hypoxic in heart failure^30^, a condition known to release ATP^31^. Given that AF-130 abolished active expiration in HF rats (Fig. 4), we predict P2X3 receptor antagonism may be effective in human HF where patients also exhibit abdominal breathing, which correlates with its severity and dyspnea^32^, yet we acknowledge that this may convey hemodynamic advantage and reduce pre-load and after-load on the left ventricle^33^.

Previously, bilateral carotid body resection improved respiratory stability in HF animals^9,28,34^. In contrast, it worsened blood gas desaturations during periods of sleep apnea in some HF patients^12^; however, in these patients, sympathetic activity was reduced, and both exercise tolerance and quality of life improved^12^. We surmise that an absence of carotid bodies in HF may exacerbate clinical problems of sleep-disordered breathing or become threatening to those at high altitude or long haul flights when ambient oxygen partial pressures are low. Hence, a major impetus for the present study was to assess whether reducing carotid body hyperexcitability while preserving its physiological function through systemic P2X3 receptor antagonism was a potential novel strategy to control pathological breathing in chronic HF rats. All told, our data support the carotid body chemoreceptors and ATP being major mediators of the breathing disorder in HF^8,9,12,28,35^.

In addition to correcting the respiratory disturbances, focal P2X3 receptor antagonism within the carotid body also restored autonomic balance (i.e. reducing sympathetic and raising cardiac vagal tone) in the in situ preparation; this was also the case when the drug was given systemically in conscious rats. Clinically most relevant were the findings that systemic chronic treatment of P2X3 receptor blockade in HF rats: (i) prevented the deleterious progression of HF by improving cardiac output substantially; (ii) lowered classic HF biomarkers; (iii) reduced systemic inflammation; (iv) lowered heart weight, indicating that the treatment attenuated hypertrophy/compensatory myocardial pathological remodeling; (v) prevented pulmonary edema as lung weight was normalized, presumably due to improved left heart pump function; (vi) increased cardiac vagal tone and respiratory sinus arrhythmia reflecting improved central baroreceptor transmission in face of attenuated carotid body afferent drive^36^. The present findings support the notion that P2X3 receptors, especially those within the carotid body, play a pivotal role in afferent-driven pathological mechanisms of cardiovascular and respiratory diseases. In our studies where the antagonist was given systemically, we cannot rule out contributions of P2X3 receptors located on other afferent systems (e.g. cardiac^37^, skeletal muscle sensors^38^) to the aforementioned comorbidities associated with HF.

Although fibrosis of the left ventricle remained unchanged, ejection fraction improved when P2X3 receptors were antagonized systemically. This may reflect: (i) the observed improvement in wall movement perhaps due to reinstatement of ‘dormant’ tissue lying outside the epicentre of the infarct; (ii) improved coronary blood flow consequent of elevated cardiac vagal and reduced sympathetic activity; (iii) reduced sympathetic vasoconstrictor tone lowering after-load; (iv) blockade of P2X3 receptors on the heart^39^; (v) the reduced inflammatory response after P2X3 receptor blockade^40^, and; (vi) recently demonstrated cardiac pump improvement with reinstatement of respiratory sinus arrhythmia in HF^41,42^.

Several studies have confirmed the importance of immune system activation in the progression of HF in patients, and B cells and IL-1β have emerged as promising targets to treat HF^43–47^. The cellular components of the immune response in chronic HF include macrophages, T, B, mast, natural killer and dendritic cells^48^. We observed reduced abundance of plasma natural killer, pro-inflammatory T and B cells, and reduced IL-1β with chronic treatment of the P2X3 receptor antagonist in chronic HF rats versus vehicle controls. Reduction in inflammation/pro-inflammatory cytokines is relevant as these can increase carotid body type I cell excitability^49,50^: IL-1β increased carotid nerve chemosensory discharge in anesthetized rats^51^. Indeed, carotid body glomus type I cells express IL-1 receptor type I^52^. Also anti-inflammatory treatment reduced carotid body afferent discharge induced by chronic intermittent hypoxia^53^. Notably, activation of the sympathetic nervous system can activate macrophages, T and B cells in chronic HF^48^. The combination of these factors may contribute to the development of a vicious cycle whereby aberrant carotid body activity induces elevated sympathetic activity to augment inflammatory responses. Hence, P2X3 receptor antagonism may prevent this vicious cycle, reducing immune cell activation as observed herein.

Despite the current advances in the treatment of heart diseases, mortality rates in patients with HF diagnose are still high, 50% of the patients with chronic HF may die in 4 years from diagnosis, while 50% with severe HF are likely to die within 1 year^54^. Although current medication slows progression, there is no cure for HF. Patients with chronic HF that exhibit high ventilatory responses to hypoxia (i.e. high chemoreflex sensitivity) have greater mortality compared to chronic HF patients that display normal chemoreflex drive^29,55^. Logically, the carotid body could be a target to treat HF. We have demonstrated that antagonism of the upregulated P2X3 receptors in the chemoreflex afferent pathway improves heart function in HF and abolished the associated breathing disturbances. Our current findings support that P2X3 receptors are a potentially valuable therapeutic target to meet the clinical need of improving the quality of life, morbidity, and mortality in HF patients.

## Methods

### Animals

The study complied with all relevant ethical regulations. The Institutional Ethics Committee in Animal Experimentation-CEUA of the Ribeirão Preto Medical School, University of São Paulo approved the experimental protocols (Protocol number 033/2017). The experiments were carried out in adult (7-8 weeks old) and juvenile (4 weeks old) male Wistar rats supplied by the Animal Facility of the Ribeirão Preto Medical School, University of São Paulo, Ribeirão Preto, Brazil. The animals were housed under standard conditions with 24 h free access to food and water, on a 12 h light 12 h dark cycle.

### Experimental heart failure

HF was induced by myocardial infarction as described previously by us^56^. Briefly, animals were anesthetized with ketamine (50 mg/kg, im; União Química Farmacêutica Nacional S/A, Embu-Guaçu, SP, Brazil) and Xylazine (10 mg/kg im; Hertape Calier Saúde animal S/A, Juatuba, MG, Brazil), submitted to orotracheal intubation and ventilated mechanically (Advanced Safety Ventilator, Harvard Apparatus, MA1 55-7059, Holliston, MA, USA). The depth of anesthesia was assessed frequently by a noxious pinch to the tail or a paw to check for a withdrawal response. Supplemental doses of anesthesia were given as required. The heart was exposed by an incision in the third intercostal space, and the anterior descending branch of the left coronary artery was identified and ligated with a silk suture (4-0). Sham rats underwent a similar surgical procedure but without coronary ligation.

### In situ working heart–brainstem preparation

These experiments could not be performed blind because the heart is exposed and visualized in the preparation. Juvenile male Wistar rats, 4 weeks, weighing 40–60 g were anesthetized deeply with isoflurane (Baxter Hospitalar, São Paulo, SP, Brazil, 5% induction, maintenance 1.5–3%) and submitted to myocardial infarction as described above. The depth of anesthesia was assessed frequently by a noxious pinch to the tail or a paw to check for a withdrawal response. Supplemental doses of anesthesia were given as required. Ten days later, rats were anesthetized deeply using isoflurane (5%), such that breathing was depressed and there was no withdrawal response to a noxious pinch to the tail or a paw, and were prepared as originally described^57^. In brief, rats were bisected below the diaphragm and made insentient via decerebration at the pre-collicular level. The carotid body and petrosal ganglion were isolated on the right side of the preparation. Preparations were transferred to a recording chamber, and a double lumen catheter was placed into the descending aorta for retrograde perfusion with a Ringer solution containing in mM: NaCl (125), NaHCO3 (24), KCl (3), CaCl_2_ (2.5), MgSO_4_ (1.25), KH_2_PO_4_ (1.25), D-glucose (10), and an oncotic agent (1.25% polyethylene glycol, Sigma), saturated with 95% O_2_−5% CO_2_ (pH, 7.35-7.4) and warmed to 31 °C. Activation of the chemoreflex was evaluated by administration of potassium cyanide (KCN; 0.05 mL, i.v., 0.05%)^57^. A neuromuscular blocking agent (vecuronium bromide, 3-4 μg/mL, Cristália Produtos Químicos Farmacêuticos) was added to prevent respiratory-related movement. Recordings from the PN, tSN, and AbN were made simultaneously using custom bipolar glass suction electrodes. The activity of the tSN was recorded from levels T8-T12 and AbN at the thoraco–lumbar level. HR was derived from the inter R wave of the ECG. All signals were amplified (10X), band-pass filtered (1700 amplifier; A-M Systems, Sequim, WA, USA; 0.1 Hz–5 kHz), and acquired (5 kHz) with an A/D converter (CED 1401, Cambridge Electronic Design, CED) controlled by a computer running Spike 2 software (Cambridge Electronic Design, CED). The noise level from the sympathetic chain was measured after the application of lidocaine (2%) at the end of each experiment and subtracted. All nerves were recorded in absolute units (μV), and analyses were performed off-line. Signals were rectified and integrated (50 ms time constant). Whole-cell current clamp recordings (Axopatch-200B integrating amplifier; Molecular Devices) of chemoreceptive petrosal neurons were performed^18^ using electrodes filled with a solution containing in mM: K-gluconate (130), MgCl_2_ (4.5), trisphosphocreatine (14), HEPES (10), EGTA (5), Na-ATP (5), Na-GTP (0.3). This solution had an osmolarity of ~ 300 mOsmol/Kg.H_2_O, pH 7.39, and resistance of 6–8 MΩ. Electrodes were positioned into the petrosal ganglion along its lateral aspect using a micromanipulator (PatchStar; Scientifica, Uckfield, UK) under visual control (microscope; Seiler, St Louis, MO, USA). The chemoreceptive petrosal neurons were functionally identified by their excitatory response to KCN^18^. The signals were amplified (10X), filtered (low pass filter 2 kHz), and acquired (10 kHz) with an A/D converter (Axon Digidata 1550B; Molecular Devices) controlled by a computer running pClamp software (Molecular Devices).

Baseline PN activity was assessed by burst frequency (Hz). To perform comparisons of the tSN recordings between groups, changes in activity were expressed as percentage changes in accordance with a scale (0–100%) determined for each preparation, as previously described^58,59^. Briefly, the maximal level of tSN produced by carotid body stimulation was used as 100%. Respiratory sinus arrhythmia was evaluated by the peak-to-trough difference in HR between inspiration and expiration. The tSN (averaged across all respiratory phases and during expiration only) and AbN expiratory responses to KCN was assessed by the measurement of the area under the curve and expressed as percentage values relative to baseline (Δ tSN and Δ AbN in percentage). PN response to KCN was assessed by the difference between baseline PN frequency and the peak of response observed after the KCN (Δ PN in Hz). Rat groups included: Sham coronary ligation, Sham + AF-353 injected into the carotid bodies, HF and HF + AF-353 injected into the carotid bodies. The electrophysiological properties of petrosal neurons measured were: (a) baseline membrane potential; (b) baseline firing frequency, and; (c) firing response to chemoreflex activation. The baseline membrane potential was assessed using a cumulative histogram (bin width 0.5 s) from the membrane potential recordings. Their firing response to chemoreflex activation was assessed by the difference between baseline firing frequency and the peak of response observed after KCN. Note: carotid body excitability is defined as either the level of carotid sinus nerve activity recorded at baseline (after background has been subtracted) or the change in carotid sinus nerve activity to stimulation with KCN. Carotid body/chemoreflex hyperreflexia refers to the magnitude of the reflex evoked response in tSN or PN activities.

### Chronic AF-130 treatment

Graphic timeline of the experimental protocol is displayed in the supplemental material (Fig S9). Either AF-130 administration (Afferent Pharmaceuticals, San Mateo, California, USA), 30 mg/kg s.c. per day or vehicle (dimethylsulfoxide 99.9%, DMSO, Sigma-Aldrich, St. Louis, MO, USA) started three days after myocardium infarction surgery and lasted for 7-8 weeks. Rat groups included: sham coronary ligation treated with vehicle (Sham), CHF treated with vehicle (CHF + vehicle) and CHF treated with AF-130 (HF + AF-130).

### Respiratory and blood gases measurements in conscious rats

The femoral artery was catheterized 24 h before the arterial blood gases measurements. Rats were anaesthetized with ketamine and xylazine and a catheter was inserted into the femoral artery, directed to the abdominal aorta (PE-10 connected to PE-50 tubing; Clay Adams, Parsippany, NJ, USA). The depth of anesthesia was assessed frequently by a noxious pinch to the tail or a paw to check for a withdrawal response. Supplemental doses of anesthesia were given as required. Samples of arterial blood (100 μl) were collected using the femoral catheter before and during the animals’ respiratory irregularities to analyze the PaCO_2_ and PaO_2_ (gas analyzer; Cobas b121; Roche Diagnostics GmbH, Germany).

Tidal volume (V_t_), respiratory rate (RR), and minute ventilation (V_E_) were studied by whole-body plethysmography in conscious rats. Pressure oscillations caused by respiratory movements were detected by a differential pressure transducer (ML141, ADInstruments, Sydney, Australia) and were digitally recorded in an IBM/PC connected to a PowerLab System (ML866, ADInstruments, Sydney, Australia). V_t_ was calculated using the formula described by Bartlett and Tenney^60^. RR was calculated from the excursion of the V_t_ signal using the cyclic rate built into the computer software LabChart v7.2 (ADInstruments, Sydney, Australia). V_E_ was calculated as the product of V_t_ and RR. Breathing interval variability was assessed from resting breathing recordings by Poincaré plots and analysis of SD1 and SD2^61^. Apnea and hypopnea incidence, considered as cessation (for a period greater than a control respiratory cycle length at rest) or 50% reduction in V_t_ over 3 consecutive breaths, were calculated and reported as apnea and hypopnea index (events/h). Post-sigh apneas numbers were also measured.

### Chemoreflex function and respiratory measurements in anesthetized animals

Animals were anesthetized with urethane (1 g/kg, i.p., Sigma Chemical, St. Louis, MO) and the depth of anesthesia was assessed frequently by a noxious pinch to the tail or a paw to check for a withdrawal response. Supplemental doses of anesthesia were given as required. Rats were placed on a heating pad (ALB 200 RA; Bonther, Ribeirão Preto, Brazil), and core body temperature maintained at 37 °C via a heating blanket with feedback from a rectal thermocouple (MLT1403; Harvard Apparatus, Holliston, MA, USA). A polyethylene catheter (Intramedic, Clay Adams, Parsippany, NJ) was inserted into the femoral vein. The carotid bifurcation was exposed and the carotid sinus nerve isolated, as we previously described^57^. Briefly, the carotid sinus nerve was traced from its point of convergence with the glossopharyngeal nerve and traced caudally towards the common carotid artery bifurcation. All measurements were performed in spontaneous breathing animals with or without the trachea cannulated breathing room air and vagus nerves intact. Teflon-coated bipolar stainless steel electrodes were implanted in the Dia and the Abd muscles for EMG recordings^62^. Activation of the chemoreflex was evaluated by administration of KCN (0.05 mL, i.v., 0.05%)^58^. All recorded signals were amplified (10X; 1700 amplifier; A-M Systems, Sequim, WA, USA), band-pass filtered (0.3 Hz – 5 kHz), and acquired by a data acquisition system (5 kHz; ML866; ADInstruments) controlled by a computer running LabChart software (v.5.0; ADInstruments). The recorded signal from the carotid sinus nerve was fed to a spike amplitude discriminator and counter, which digitally counted in 1 s intervals to assess its discharge frequency (spikes per second). Changes in carotid sinus nerve in response to KCN were assessed by the difference between baseline and the peak of response observed after the stimulus (Δ CSN). EMGs were recorded in absolute units (μV) and analyses were performed off-line from rectified and integrated (∫) signals (time constant: 50 ms). Dia_EMG_ burst frequency was assessed as RR. Changes in the Abd_EMG_ activity during baseline condition were expressed in µV. Based upon absolute values, we determined percentage changes in order to compare their activities in each animal. At the end of the experimental procedures, blood samples were collected for further analysis of plasma N-Terminal Pro-B-Type natriuretic peptide (NT-proBNP; see below) concentration. Rats were euthanized with a high dose of pentobarbital (100 mg/kg, i.v.) and once breathing had ceased the lungs and hearts were removed, rinsed in ice-cold 0.9% NaCl solution, dried, and weighed. The heart was fixed in 3.7% formaldehyde, embedded in paraffin, and the sections were stained with Masson’s trichrome to reveal the infarct size and measured using the NIH ImageJ software (developed by National Institutes of Health and available on the internet site http://rsb.info.nih.gov/nih-image/). Infarct size was calculated by dividing the length of the infarcted area by the total circumference of the LV and expressed as a percentage^56^.

### Echocardiography

The echocardiographic evaluation was performed one day before the myocardial surgery (control), and repeated three days and seven weeks after the myocardial infarction in chronic HF rats. In juvenile rats, the echocardiographic analysis was performed ten days after the myocardial infarction. Rats were anesthetized with ketamine (50 mg/kg) and Xylazine (10 mg/kg, i.m.), and the depth of anesthesia was assessed frequently by a noxious pinch to the tail or a paw to check for a withdrawal response. Supplemental doses of anesthesia were given as required. Body temperature was monitored and maintained, and cardiac parameters were obtained through a VEVO2100® (Fuji) machine using a 30 MHz transducer. Diastolic left ventricle diameter, and ventricular posterior wall thickness were evaluated in M-mode; end systolic volume, stroke volume, and ejection fraction were calculated using a bidimensional mode.

### Analysis of R-R wave interval variability

The rats were anesthetized transiently with isoflurane (Baxter Hospitalar, São Paulo, SP, Brazil, 5% induction, maintenance 1.5–3%) and subcutaneous electrocardiogram (ECG) electrodes were implanted. After 48 h, the ECG signal was recorded for 1 h in the conscious state. R-R wave interval variability analysis was performed in the frequency domain using CardioSeries software (v2.7, www.danielpenteado.com). The R-R interval time series were resampled at 10 Hz (1 value every 100 ms) by cubic spline interpolation, to regularize the time interval between beats. The R-R interval time series with 15 min duration were divided into 34 half-overlapping (Welch protocol) segments, each one with 512 values. Next, Hanning windowing was employed and each stable segment was subjected to spectral analysis using Fast Fourier Transform. Pulse interval spectra were integrated into low (LF: 0.20–0.75 Hz) and high frequency (HF: 0.75–3.00 Hz) frequency bands. LF and HF powers are expressed in normalized units (nu) and the LF/HF ratio is also shown.

### NT-proBNP analysis

Plasma NT-proBNP concentration was measured using AssayMax™ immunoenzymatic assay kit following the manufacturers instructions (St. Charles, MO, USA, catalogue number: ERB1202-1).

### RT–qPCR

In the single-cell RT-qPCR experiments, the pipette solution containing the cytoplasmatic material of the recorded petrosal neuron was collected from the patch pipette. The High Capacity cDNA Reverse Transcription Kit reagents (Life Technologies) and nuclease-free water were used for subsequent transcription in a thermocycler (ProFlex PCR System; Applied Biosystems, Foster City, CA, USA). cDNA pre-amplification was performed in the single-cell RT-qPCR experiments using the TaqMan PreAmp Master Mix Kit (Life Technologies) using the P2X2 (Rn04219592_g1), P2X3 (Rn00579301_m1) and β-actin (NM_031144.2) probes. The reactions for the RT-qPCR were performed in singleplex and triplicate (StepOnePlus System, Applied Biosystems) using the same probes described above and the TaqMan Universal PCR Master Mix kit (Life Technologies) according to the manufacture’s recommendations. β-actin was used as a house keeping control gene to normalize reactions. The relative quantitation was determined by the ΔΔCt method. For each sample, the threshold cycle (Ct) was determined and normalized relative to β-actin (ΔCt = Ct Unknown – Ct referencegene). The fold change of mRNA content from the petrosal ganglia chemoreceptive neurons from HF relative to the sham animals was determined by 2 − ΔΔCt, where ΔΔCt = ΔCt Unknown – ΔCt Control. Data are presented as mRNA expression relative to the sham animals.

### Immunocytochemical studies

Carotid bifurcations from HF rats were surgically removed immediately after the in situ experiments and transferred into ice cold Ringer. Carotid bodies were dissected, fixed overnight with 4% formaldehyde, and submerged in sucrose solution (30%) for 24 h. Coronal sections (40 μm thick) were washed three times in phosphate-buffered saline (PBS 0.1 M) for 5 min and then blocked and permeabilized in PBS, 10% normal horse serum, and 0.1% Triton X-100 for one hour (room temperature). The sections were incubated in mouse anti-tyrosine hydroxylase (TH; 1:1000; Millipore, Burlington, MA, USA) and in rabbit anti-P2X3 receptor (1:500; Abcam, Waltham, MA, USA) primary antibodies overnight. In sequence, they were washed three times with PBS for 5 min, followed by incubation in goat anti-mouse Alexa Fluor 488 (1:500; Thermo Fisher Scientific, Waltham, MA, USA) and goat anti-rabbit Alexa 647 (1:500; Thermo Fisher Scientific) for 4 h. We performed negative controls to show an absence of non-specific staining from secondary antibodies (Fig S12). Subsequently, sections and cells were mounted onto glass slides with Fluoromount (Sigma-Aldrich). The images were acquired using a Leica TCS SP5 (Wetzlar, Germany) confocal microscope equipped with 488 and 633 nm lasers and detection of tunable emission wavelengths.

### Infarct size analysis

The hearts were fixed in phosphate-buffered 4% formalin and mounted in paraffin blocks. Each block was serially cut at 6 μm from the midventricular surface. The sections were stained with Masson’s trichrome, and the infarct size was measured using the NIH ImageJ software (developed by the National Institutes of Health; http://rsb.info.nih.gov/nih-image/). Infarct size was calculated by dividing the length of the infarcted area by the total circumference of the LV and expressed as a percentage^56^.

### Inflammatory cells

In this protocol, rats were submitted to myocardial infarction and the administration of vehicle (DMSO) or AF-130 (2 mg/kg/h) started four weeks after the surgical procedure. For vehicle or AF-130 infusion, a polyethylene catheter was inserted in the jugular vein and connected with a programmable iPRECIO SMP-300 pump (Primetech Corporation, Tokyo, JP) placed under the skin of the back. The animals were treated for 3 weeks. At the end of the treatment, blood samples were collected from the tail vein. The cells from the experimental groups were placed in 96-well round-bottom plates for cytofluorometric analysis. Following Fc receptor blocking, cells were incubated with colour combinations of the monoclonal antibodies (BD Biosciences, San Jose, CA, USA). Stained cells were stored for analysis in PBS containing 1% paraformaldehyde, in sealed tubes held in the dark. All steps were performed at 4 °C. Analysis of these cells was performed using a Becton Dickinson FACScan flow cytometer with DIVA-BD software (Becton Dickinson Immunocytometry Systems, San Jose, CA, USA). Representative plots of gating strategy are showed in figures S13 and S14.

### Cytokine measurements

Plasma cytokine (TNF-α, IL-1β, and IL-10) levels were analyzed by the immune-enzymatic ELISA method, using Duo set kits (R&D Systems, Minneapolis, MN, USA) according to the manufacturers’ instructions.

### Drugs

Two antagonists with very similar P2X3 and P2X2/3 selectivity were used. AF-353 (Afferent Pharmaceuticals) has a low polar surface area and as a result, crosses the blood-brain barrier^63^, however AF-130 (Afferent Pharmaceuticals), with a methyl sulfone substitution (Supplementary Fig. S10, making the overall selectivity/affinity profile similar, has a much higher polar surface area, and does not cross the blood-brain barrier^64,65^. The latter was used in the in vivo studies. AF-130 data were generated by Afferent Pharmaceuticals, are unpublished and include that this antagonist is a highly selective and potent inhibitor of P2X3 and P2X2/3 channels showing greater potency at P2X3 homotrimers than P2X2/3 heterotrimers by around eight-fold. The potency of AF-130 is reflected by the IC50 ranges of 126–407 nM for P2X3 receptors and 240–5670 nM for P2X2/3 receptors. AF-130 has >25-fold selectivity over other P2X channels tested (including P2X1, P2X2, P2X4, P2X5 and P2X7). It has been tested on 73 non-purinergic targets (e.g, ion channels, GPCR, transporters, and enzymes). Only when doses were 25–100 fold above the IC50 range for P2X3 and P2X2/3 receptors was a partial (20%) antagonism of some tested processes observed (e.g. adenosine 3 receptors, 5-HT6 receptors, and dopamine transporter). See Supplementary Figure 10 for additional information on AF-130.

### Statistical analysis

Results are expressed as the mean ± standard deviation (SD). Data were tested for normality using Kolmogorov–Smirnov test and compared using unpaired *t*-test with Welch’s correction, One-way ANOVA or repeated measures two-way ANOVA, with Student-Newman-Keuls, Bonferroni or Tukey *post hoc* comparisons. Correlations were assessed using Pearson’s correlation coefficients. The type of statistical test performed is indicated in the figure legends. Differences were considered to be statistically significant with *p* < 0.05.

### Reporting summary

Further information on research design is available in the Nature Portfolio Reporting Summary linked to this article.

## Supplementary information

Supplementary Information

Reporting Summary

## Data Availability

All data which supports the findings here can be found in the manuscript and supplementary information. Source data for all experiments are provided with this study. Source data are provided with this paper.

## Source data

Source Data
