# Supplementary material for: P2X3 receptor antagonism attenuates the progression of heart failure
Source: Nat Commun. 2023 Mar 28;14:1725. doi: 10.1038/s41467-023-37077-9 (PMC10050083; doi:10.1038/s41467-023-37077-9)
Supplement: Supplementary file 1 — Supplementary Information [file 41467_2023_37077_MOESM1_ESM.pdf]

Supplemental Figures for:

P2X3 receptor antagonism attenuates the progression of heart failure

Renata M. Lataro[1], Davi J. A. Moraes[2], Fabio N. Gava[2,3], Ana C. M. Omoto[2], Carlos A. A. Silva[2], Fernanda Brognara[2], Lais Alflen[1], Vânia Brazão[4], Rafaela Pravato Colato[4], José Clóvis do Prado Jr[4], Anthony P. Ford[5], Helio C. Salgado[2], Julian F. R. Paton[6*].

[1]Department of Physiological Sciences, Center of Biological Sciences, Federal University of Santa Catarina, Florianópolis, Santa Catarina, Brazil

[2]Department of Physiology, Ribeirão Preto Medical School, University of São Paulo, Ribeirão Preto, Brazil

[3]Department of Clinical Veterinary, Agrarian Sciences Center, Londrina State University, Londrina, Brazil

[4]College of Pharmaceutical Sciences of Ribeirão Preto, University of São Paulo, Ribeirão Preto, Brazil

[5]CuraSen, 2655 Campus Dr #110, San Mateo, CA 94403, United States

[6]Manaaki Manawa -the Centre for Heart Research, Department of Physiology, Faculty of Medical & Health Sciences, University of Auckland, Auckland, New Zealand.

Corresponding author

Professor Julian F. R. Paton

Department of Physiology

Faculty of Medical & Health Sciences

University of Auckland, Auckland, New Zealand

j.paton@auckland.ac.nz

Table S1

	Chemoreceptive neurons		Non-chemoreceptive neurons	
	**Sham**	**HF**	**Sham**	**HF**
**Resting membrane potential (mV)**	-56.6 ± 3.04	-46.3 ± 3.64****	-57.38 ± 2.62#	-59.38 ± 2.54&
**Number of cells recorded**	10	6	5	5

**** Sham Chemo vs. HF Chemo (P<0.0001)
# Sham Non-chemo vs. HF Chemo (P<0.0001)
& HF Chemo vs. HF Non-chemo (P<0.0001)

Table S1

The resting membrane potential and numbers of neurons recorded from the petrosal ganglion is shown for both those that were activated by sodium cyanide stimulation of the carotid body ('Chemoreceptive neurons') versus those that did not respond ('Non-chemoreceptive neurons').

Figure S1

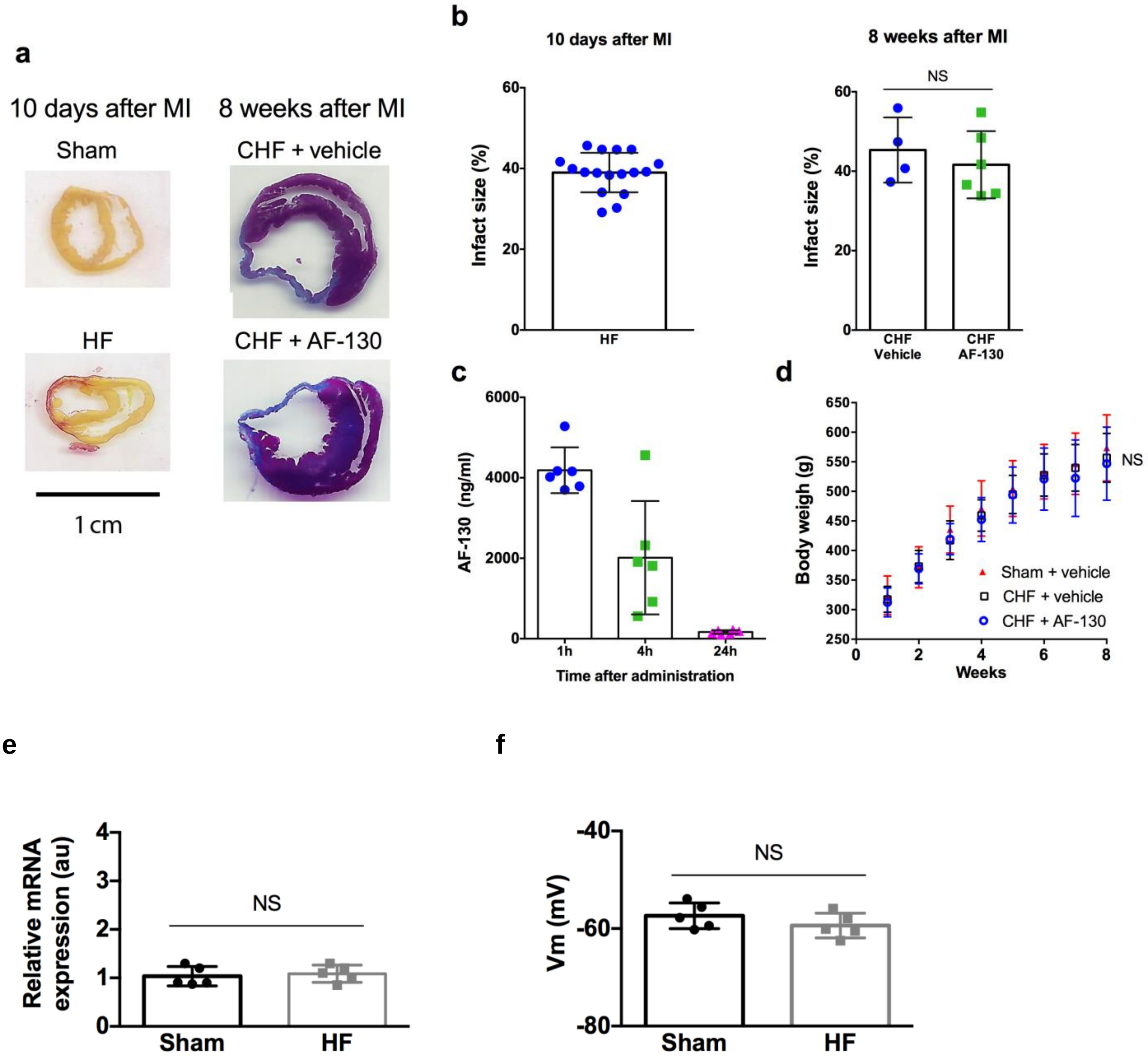

Fig S1 – Infarct size, AF-130 pharmacokinetics and body weight.

(**a**) Representative transverse section through the left ventricle of sham and heart failure (HF) rats stained with picrosirius red and trichrome after 10 days and 8 weeks after myocardial infarction (MI), respectively. (**b**) Quantitative data showing infarct size in 10 days post-MI rats (HF, *n*=17) and vehicle and drug treated rats with chronic heart failure (CHF, 8 weeks, *n*=4 and 6 for CHF vehicle and CHF AF-130 group, respectively.); these infarct

sizes were not different (Unpaired t test, two-sided, $p>0.05$). (**c**) AF-130 pharmacokinetics data from blood samples that were collected from the tail vein 1, 4 and 24 h after AF-130 administration (30 mg/kg, s.c.; *n*=6). (**d**) The age dependent increase in actual body weight between the three rat groups was not different over the 8 weeks protocol (Two-way Repeated Measures ANOVA; *n*=9). Grouped data from single non-chemoreceptive petrosal neurons depicting (**e**) P2X3 receptor mRNA expression and resting membrane potential (**f**) in heart failure (HF) *versus* sham animals. Data are mean ± SD. Data were compared using Unpaired t test, two-sided; n=5 per group in (**e**) and (**f**); ns: not significant. Source data are provided with this paper.

Figure S2

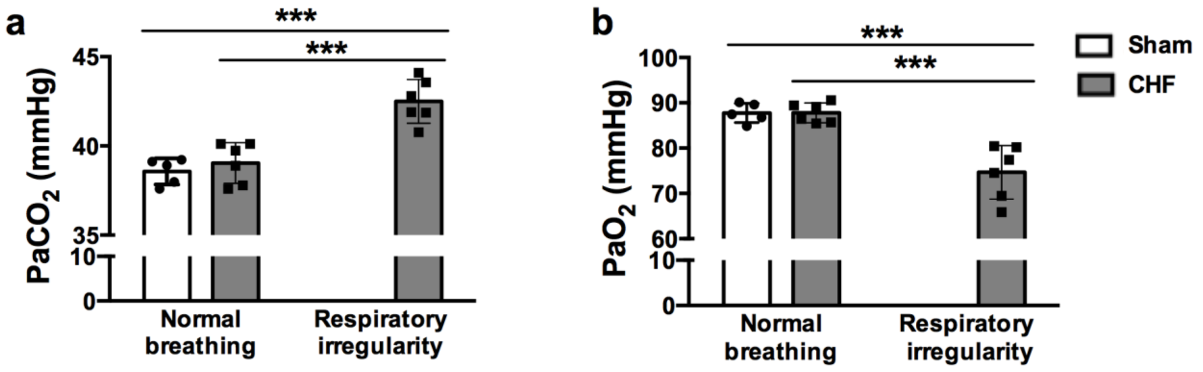

Fig S2 - Blood gas data.

The arterial blood partial pressure of carbon dioxide ($PaCO_2$, **a**) and oxygen (PaO_2, **b**) in sham and chronic heart failure (CHF) rats during normal breathing and immediately after a bout of respiratory irregularity, which was seen in CHF rats only. These data support the contention that the hypercapnia and hypoxia were a consequence of the breathing disturbance, but did not cause it. Data are mean ± SD. Two-way ANOVA Bonferroni post test; *n*=5 and 6 for sham and CHF group, respectively. ***$P<0.001$. Source data are provided with this paper.

Figure S3

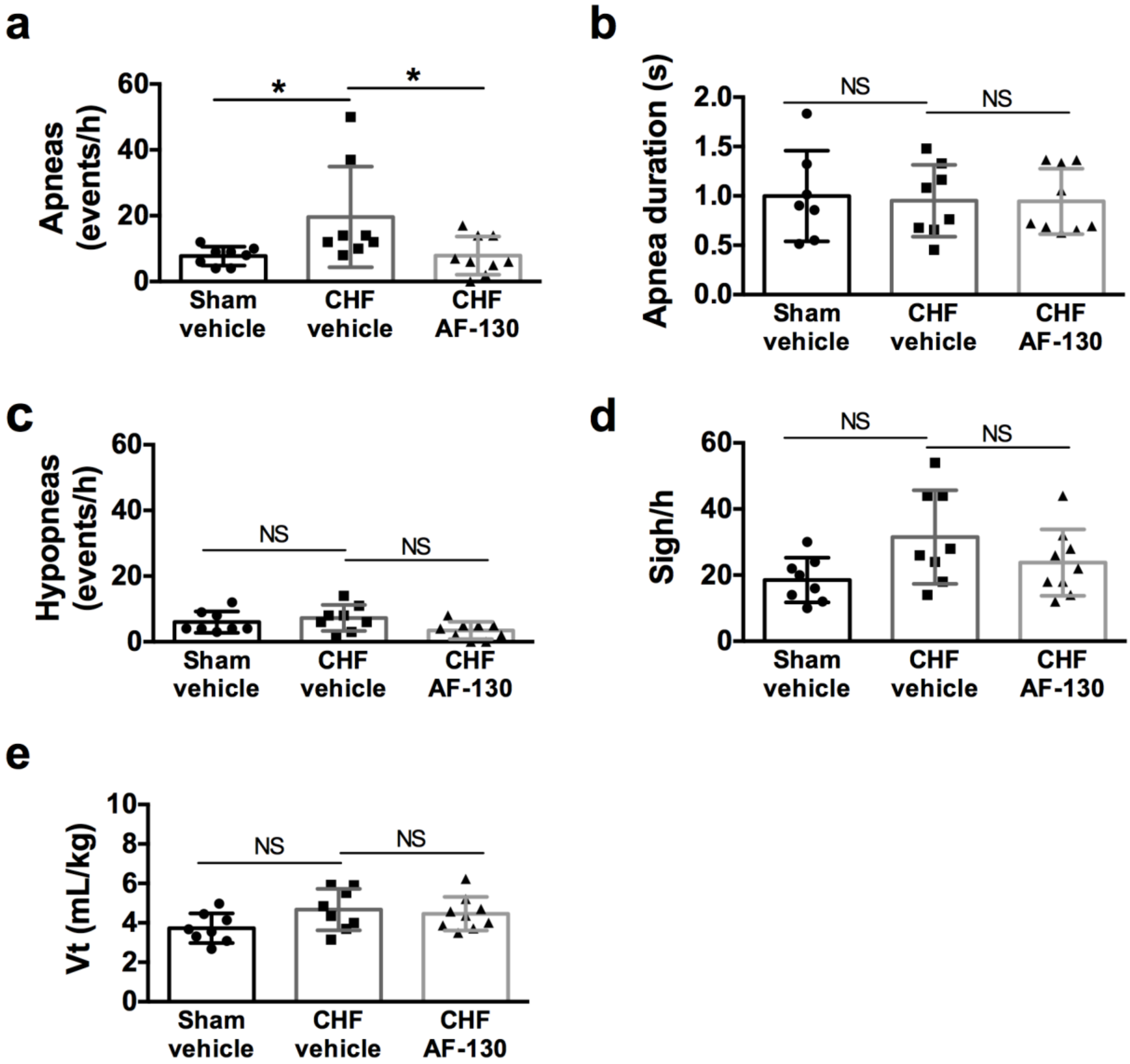

Fig S3 - Respiratory variable analyses.

The number (**a**) and duration of apneas (**b**) and the frequency of occurrence of the hypopneas (**c**) are shown separately. Also indicated are the numbers of sighs per hour (**d**) and tidal volume (V_t) (**e**). In all cases, data are compared across sham animals given vehicle (*n*=8 in a,d-e and *n*=7 in b), chronic heart failure (CHF) rats administered either vehicle (*n*=8) or AF-130 (*n*=9), the P2X3 receptor antagonist. Data are mean ± SD. One-way ANOVA Tukey post-test. *P<0.05. Source data are provided with this paper.

Figure S4

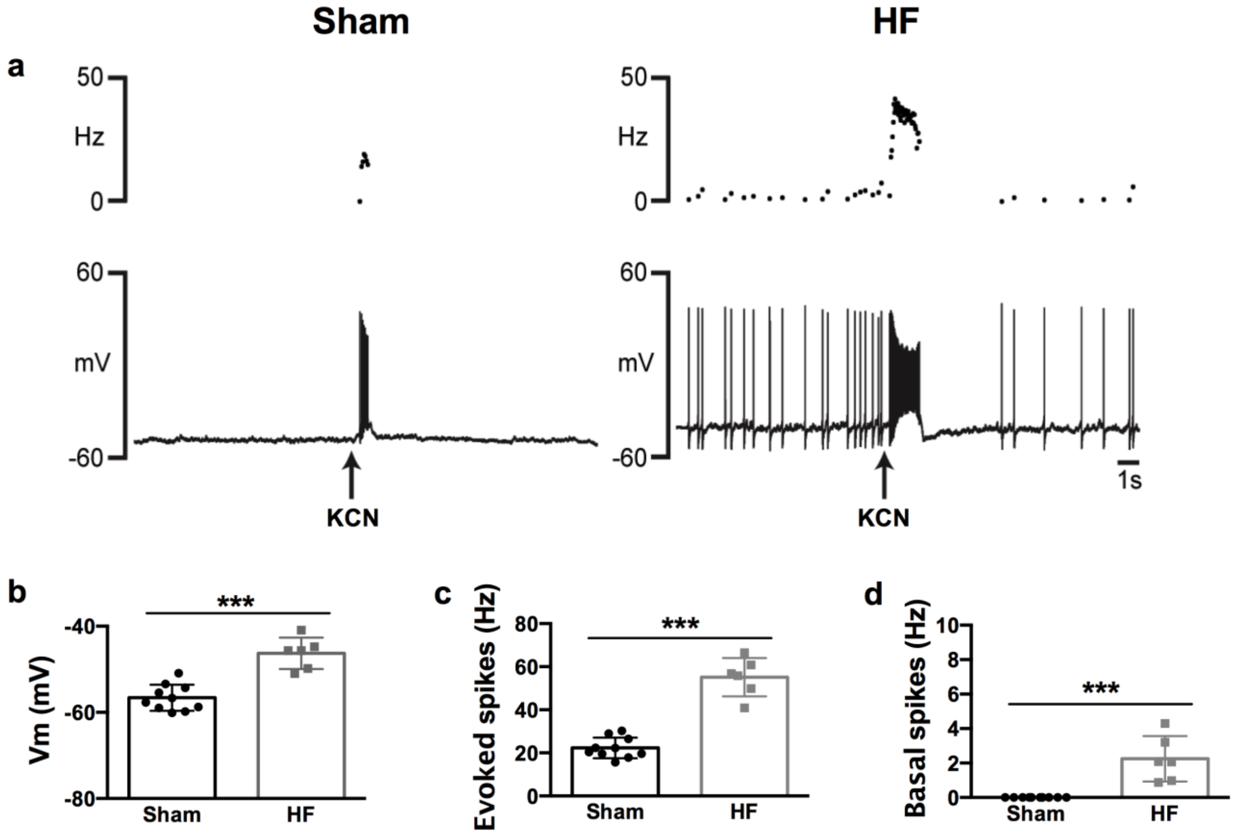

Fig S4 - Heart failure (HF) boosts electrical activity of petrosal chemoreceptive neurons.

Whole cell recordings of petrosal ganglion neurons responding to carotid body stimulation with potassium cyanide (KCN). **(a)** the firing response of a sham and HF rat demonstrating tonic activity and hypersensitivity (to KCN). Membrane potentials were depolarized in the HF rats versus shams **(b). (c)** shows the substantially larger reflex evoked firing response to KCN and **(d)** indicates that petrosal chemoreceptive neurons never showed ongoing firing in sham animals whereas a ~2 Hz firing frequency was found in all HF rats during baseline. Data are mean ± SD. Data were tested for normality (Shapiro-Wilk test) and compared using Unpaired t test, two-sided; *n*=10 and 6 for sham and CHF group, respectively. ***P<0.001. Source data are provided with this paper.

Figure S5

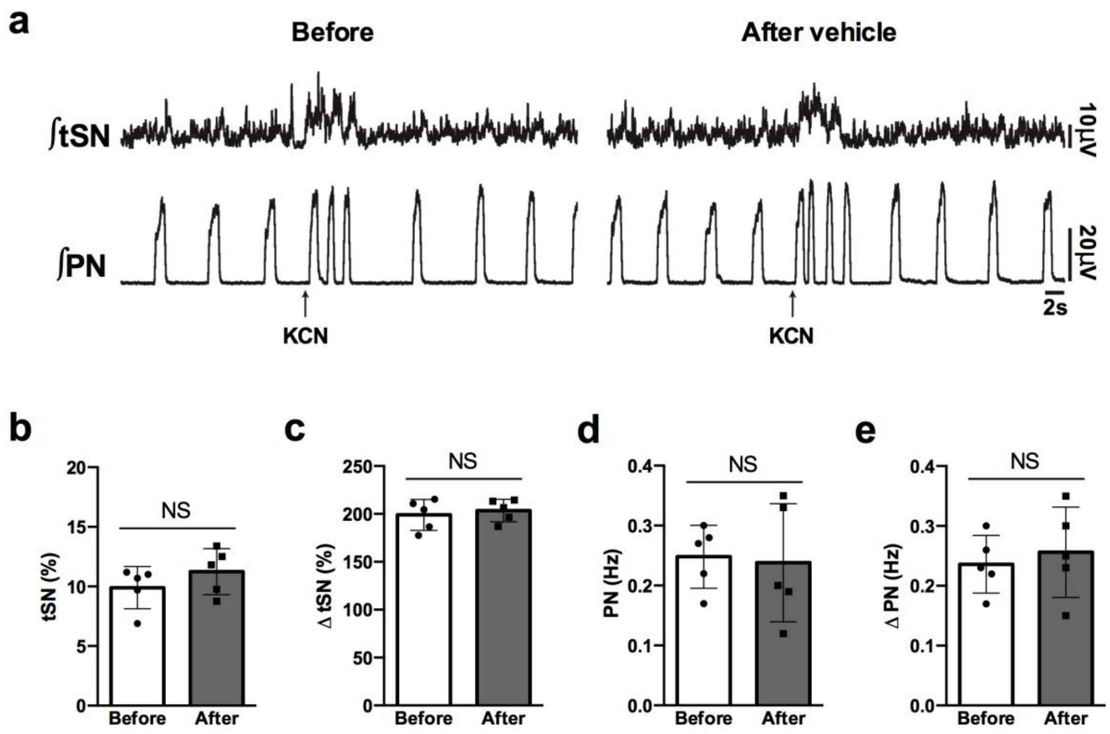

Fig S5 - Volume control infusions into the carotid body.

In some studies in the *in situ* working heart brainstem preparation, drugs were pico-infused directly into the carotid body. The raw (**a**) and mean data (**b-e**) shown above indicates that the insertion of the glass pipette and infusions did not affect the sensitivity of the carotid body as seen by an absence of change in the magnitude of both the basal and chemoreflex evoked changes in thoracic sympathetic nerve (tSN; **b,c,** respectively) and phrenic nerve (PN) integrated activities (**d,e,** respectively). Data are mean ± SD. Data were tested for normality (Shapiro-Wilk test) and compared using Paired t test, two-sided; *n*=5; ns: not significant. Source data are provided.

Figure S6

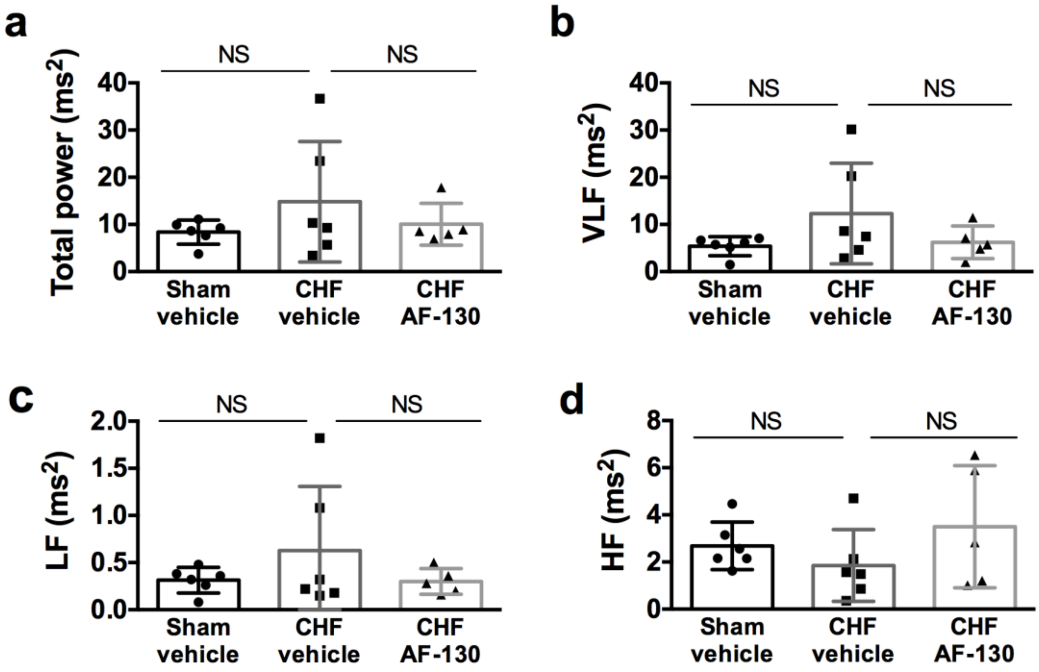

Fig S6 - Heart rate variability analysis based on absolute values from rats with chronic heart failure (CHF)

The total power (**a**), very low frequency (VLF; **b**), low frequency (LF, **c**) and high frequency (HF, **d**) for both sham animals treated with vehicle and CHF rats treated with either vehicle or the P2X3 receptor blocker (AF-130) are depicted. There was no difference between groups. However, when data are normalized by expressing as percentage change then these trends become significant. Data are shown as mean ± SD. One-way ANOVA Tukey post-test; *n*=6 for sham vehicle, *n*=6 for CHF vehicle and *n*=5 for CHF AF-130 group; ns: not significant. Source data are provided with this paper.

Figure S7

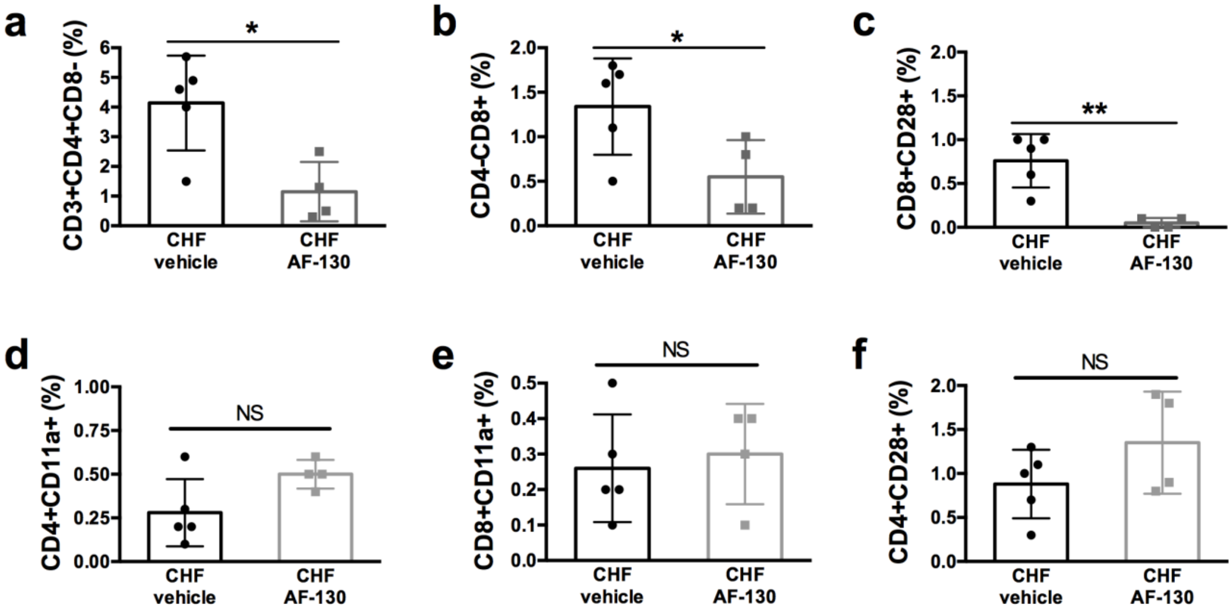

Fig S7 - Heterogeneity in the response of immune cell types to P2X3 receptor antagonism.

The ability of chronic P2X3 receptor antagonism on a number of immune cells expressing different antigens was tested in chronic heart failure (CHF, *n*=4) rats over three weeks and compared to those receiving vehicle (*n*=5; **a-f**). Note that not all immune cell types were suppressed by blocking P2X3 receptors systemically. Data are mean ± SD. Data were compared using unpaired t test with Welch's correction, two-sided. *P<0.05, **P<0.01. Source data are provided with this paper.

Figure S8

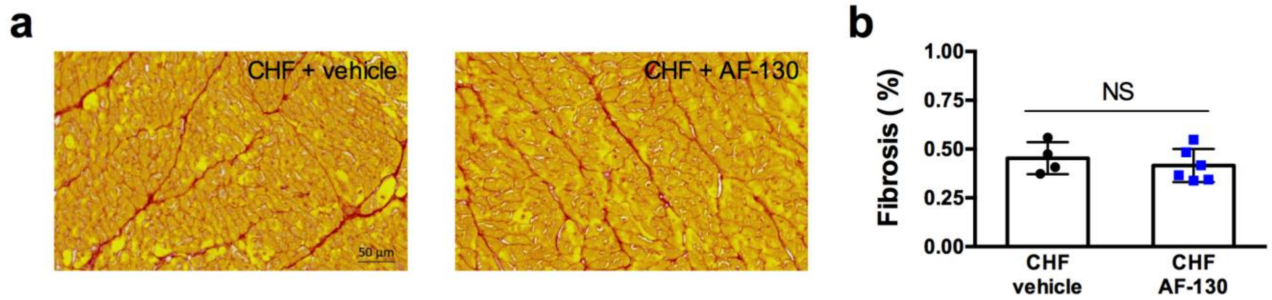

Fig S8 - Myocardial fibrosis in chronic heart failure (CHF) rats treated with vehicle or with AF-130.

(**a**) Photomicrographs and (**b**) bar graphs of the collagen density from the left ventricle of CHF rats treated with vehicle (*n*=4) or with AF-130 (*n*=6). Data are mean ± SD. Data were compared using unpaired t test, two-sided; ns: not significant. Source data are provided with this paper.

Figure S9

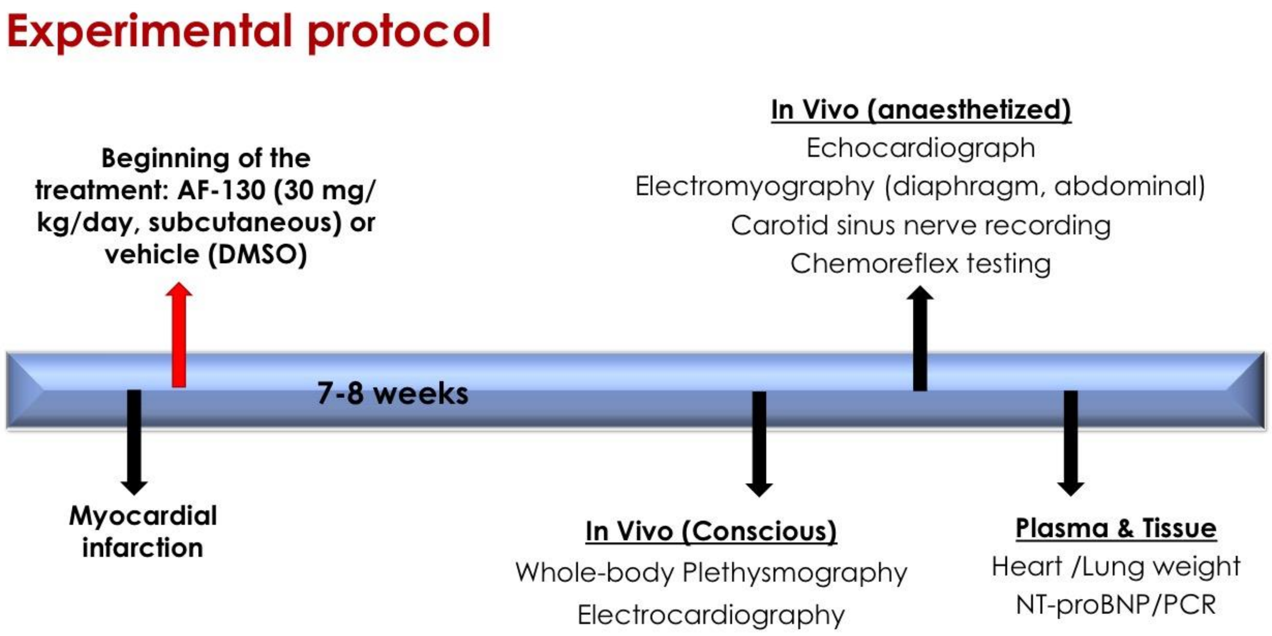

Fig S9 - Timeline of the chronic AF-130 treatment in heart failure rats.

Treatment with vehicle or AF-130 started three days after myocardium infarction surgery and lasted for 7-8 weeks. At the end of the treatment, heart rate was recorded by electrocardiography while cardiac autonomic modulation was assessed by heart rate variability, cardiac function was evaluated by echocardiography and respiration measured by whole-body plethysmography and electromyography (diaphragm, abdominal). Carotid sinus nerve recording and chemoreflex testing was also performed.

Figure S10

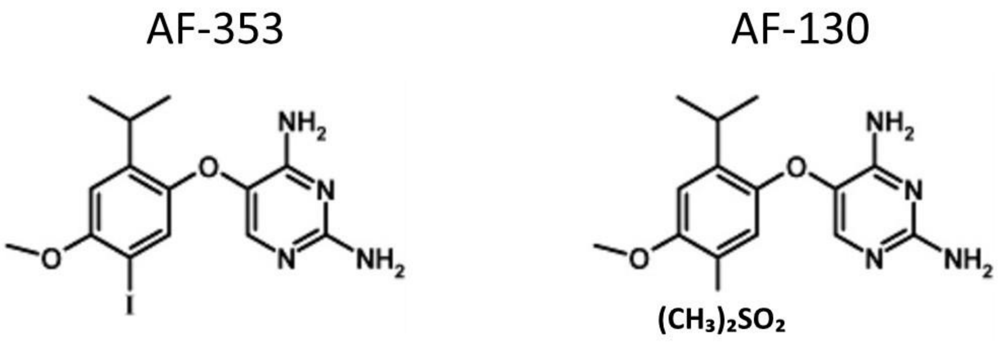

Fig S10 - Chemical structures of AF-353 and AF-130

The two P2X3 receptor antagonists used in the study indicate that the difference is a methyl sulfone substitution for iodide in the AF-130 molecule. See references 63 & 64 for further details.

Figure S11

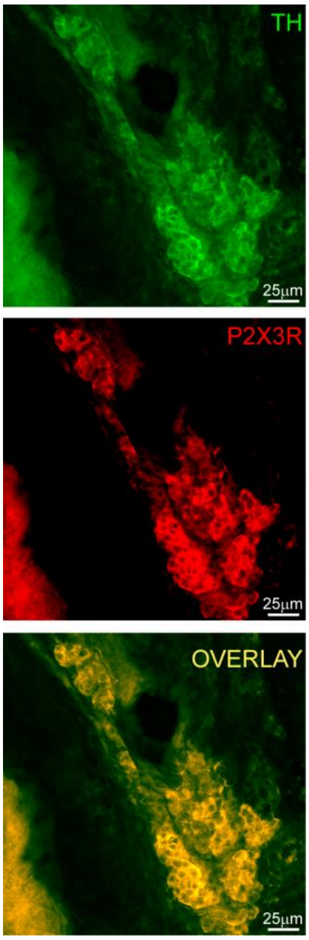

Fig S11

Immunofluorescence of P2X3 receptors within the carotid body of a heart failure rat. Note the coincidence (OVERLAY) of P2X3 receptor immunofluorescence (red) with tyrosine hydroxylase (TH, green), a marker for type I glomus cells. This was repeated in three rats. Scale bar 25 µm.

Figure S12

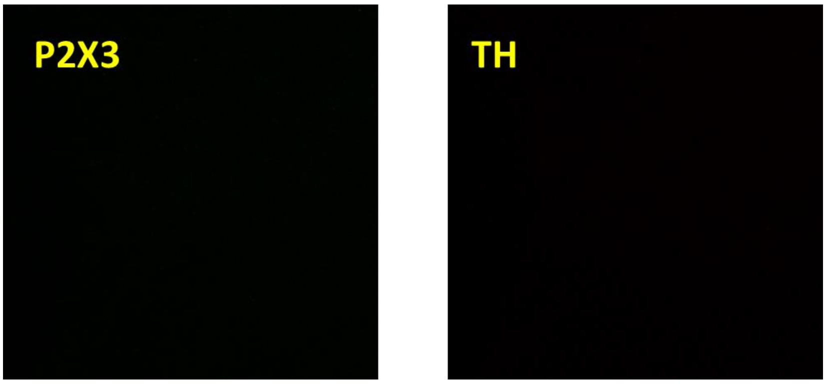

Fig S12

Negative controls for the secondary antibodies used for P2X3 receptors and tyrosine hydroxylase (TH) labelling in the carotid body of heart failure rats.

Figure S13

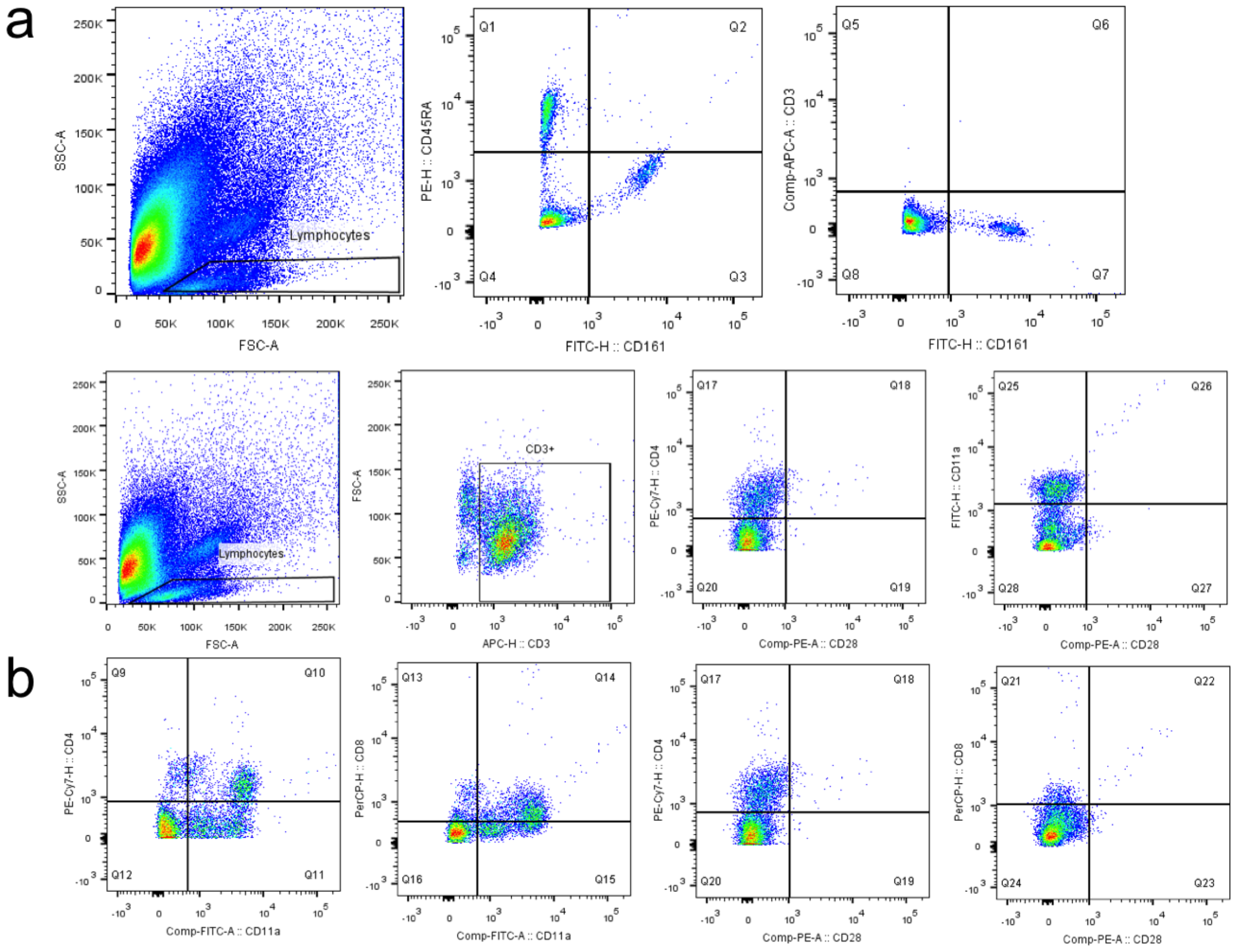

Fig S13

Dot plot representation of CD45RA$^+$ and CD161$^+$ positive cells (**a**) and (**b**) dot plot representation shows a representative example of CD11a and CD28 expression on CD3$^+$CD4$^+$ and CD3$^+$CD8$^+$ T cells.

Figure S14

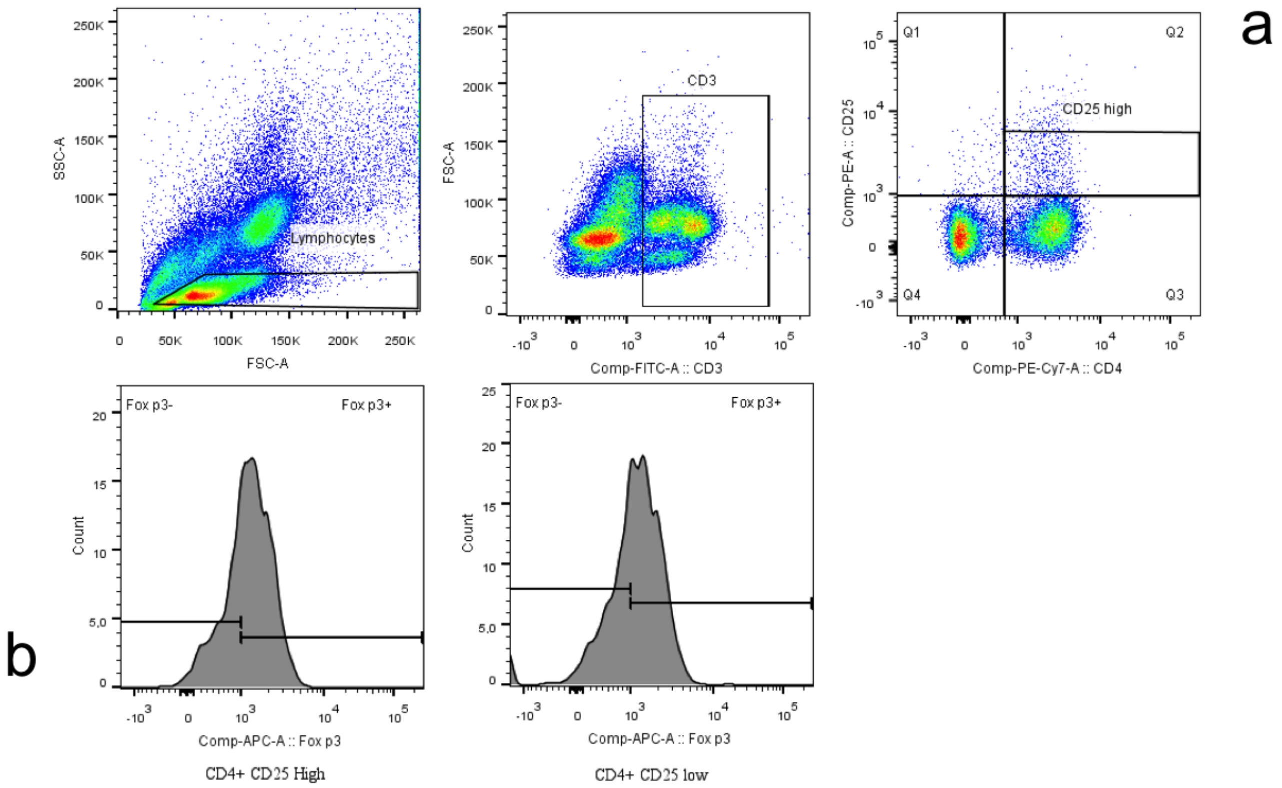

Fig S14

Representative plots of gating strategy (**a**). Dot plot representation of $CD4^{+}CD25\ hi^{+}FOXP3$ T regulatory cells. The upper gate on the dot plots (gate a) represents $CD4^{+}CD25hi^{+}$ T cells and FOXP3 expression was gated on $CD4^{+}CD25hi^{+}$ T cell population (**b**; gate b).
