# Supplementary material for: P2X3 receptor antagonism attenuates the progression of heart failure
Source: Nat Commun. 2023 Mar 28;14:1725. doi: 10.1038/s41467-023-37077-9 (PMC10050083; doi:10.1038/s41467-023-37077-9)
Supplement: Supplementary file 2 — Reporting Summary [file 41467_2023_37077_MOESM2_ESM.pdf]

nature portfolio

Corresponding author(s): Julian F. R. Paton

Last updated by author(s): Feb 28, 2023

Reporting Summary

Nature Portfolio wishes to improve the reproducibility of the work that we publish. This form provides structure for consistency and transparency in reporting. For further information on Nature Portfolio policies, see our Editorial Policies and the Editorial Policy Checklist.

Statistics

For all statistical analyses, confirm that the following items are present in the figure legend, table legend, main text, or Methods section.

n/a	Confirmed	
☐	☒	The exact sample size (*n*) for each experimental group/condition, given as a discrete number and unit of measurement
☐	☒	A statement on whether measurements were taken from distinct samples or whether the same sample was measured repeatedly
☐	☒	The statistical test(s) used AND whether they are one- or two-sided<br>*Only common tests should be described solely by name; describe more complex techniques in the Methods section.*
☐	☒	A description of all covariates tested
☐	☒	A description of any assumptions or corrections, such as tests of normality and adjustment for multiple comparisons
☐	☒	A full description of the statistical parameters including central tendency (e.g. means) or other basic estimates (e.g. regression coefficient) AND variation (e.g. standard deviation) or associated estimates of uncertainty (e.g. confidence intervals)
☐	☒	For null hypothesis testing, the test statistic (e.g. *F*, *t*, *r*) with confidence intervals, effect sizes, degrees of freedom and *P* value noted<br>*Give P values as exact values whenever suitable.*
☒	☐	For Bayesian analysis, information on the choice of priors and Markov chain Monte Carlo settings
☒	☐	For hierarchical and complex designs, identification of the appropriate level for tests and full reporting of outcomes
☐	☒	Estimates of effect sizes (e.g. Cohen's *d*, Pearson's *r*), indicating how they were calculated

Our web collection on statistics for biologists contains articles on many of the points above.

Software and code

Policy information about availability of computer code

Data collection	Spike 2 software v7 (Cambridge Electronic Design, CED)<br>LabChart v5.0 and v7.2 (ADInstruments, Sydney, Australia)
Data analysis	Spike 2 software (v2, Cambridge Electronic Design, CED)<br>LabChart v5.0 and v7.2 (ADInstruments, Sydney, Australia)<br>NIH ImageJ software (developed by National Institutes of Health and available on the internet site http://rsb.info.nih.gov/nih-image/)<br>CardioSeries software (v2.7, www.danielpenteado.com)<br>DIVA-BD software (v6.1,3 Becton Dickinson Immunocytometry Systems, San Jose, CA, USA).<br>pClamp software (v10, Molecular Devices)

For manuscripts utilizing custom algorithms or software that are central to the research but not yet described in published literature, software must be made available to editors and reviewers. We strongly encourage code deposition in a community repository (e.g. GitHub). See the Nature Portfolio guidelines for submitting code & software for further information.

Data

Policy information about availability of data

All manuscripts must include a data availability statement. This statement should provide the following information, where applicable:

- Accession codes, unique identifiers, or web links for publicly available datasets
- A description of any restrictions on data availability
- For clinical datasets or third party data, please ensure that the statement adheres to our policy

All data which supports the findings here can be found in the manuscript and supplementary information. Source data for all experiments are provided with this study.

Human research participants

Policy information about studies involving human research participants and Sex and Gender in Research.

Reporting on sex and gender	N/A
Population characteristics	N/A
Recruitment	N/A
Ethics oversight	N/A

Note that full information on the approval of the study protocol must also be provided in the manuscript.

Field-specific reporting

Please select the one below that is the best fit for your research. If you are not sure, read the appropriate sections before making your selection.

☒ Life sciences ☐ Behavioural & social sciences ☐ Ecological, evolutionary & environmental sciences

For a reference copy of the document with all sections, see nature.com/documents/nr-reporting-summary-flat.pdf

Life sciences study design

All studies must disclose on these points even when the disclosure is negative.

Sample size	Group sizes were determined by a power analysis based on previous similar studies (Pijacka, W. et al. Purinergic receptors in the carotid body as a new drug target for controlling hypertension. Nat. Med. 22, 1151–1159, 2016; O'Callaghan, E.L. et al. Enhancing respiratory sinus arrhythmia increases cardiac output in rats with left ventricular dysfunction. J Physiol 598(3):455-471, 2020), and the final sample size was adjusted for expected animal death of 20%. A total number of at least 4 animals per group was obtained.
Data exclusions	The excluded animals were those with lack of akinetic left ventricle free wall determined by ultrasound measurement three days after myocardial infarction or infarct size < 30%, confirmed by post-hoc histological analysis. For the in situ work preparations were excluded the animals that failed to show a ramp inspiratory regular pattern.
Replication	This study being multi-disciplinary was performed by multiple laboratories working independently and obtaining consistently coherent data. In all experiments in the manuscript were performed 4 or 6 times according to power calculations or when statistical significance was achieved as per our ethics approval.
Randomization	Animals from the same batch were randomly assigned in two or three groups according to the experimental protocol.
Blinding	In most cases data analysis was not blinded because data was acquired by the same investigator (e.g. electrophysiology, nerve recordings, respiratory variables). On the occasion when data was acquired by separate investigators, then analysis was blinded (e.g echocardiography and flow citometry).

Reporting for specific materials, systems and methods

We require information from authors about some types of materials, experimental systems and methods used in many studies. Here, indicate whether each material, system or method listed is relevant to your study. If you are not sure if a list item applies to your research, read the appropriate section before selecting a response.

Materials & experimental systems

n/a	Involved in the study	
☐	☒	Antibodies
☒	☐	Eukaryotic cell lines
☒	☐	Palaeontology and archaeology
☐	☒	Animals and other organisms
☒	☐	Clinical data
☒	☐	Dual use research of concern

Methods

n/a	Involved in the study	
☒	☐	ChIP-seq
☐	☒	Flow cytometry
☒	☐	MRI-based neuroimaging

Antibodies

Antibodies used

1 - Millipore - Anti-Tyrosine Hydroxylase: MAB318 (catalogue number); 2651993 (lot number); LNC1 (clone)
2 - Abcam: Anti-P2X3: Ab10269 (catalogue number); Gr168296-13 (lot number)
3- Life Technologies - goat anti-mouse Alexa Fluor 488: A10680 (catalogue number); 1613049 (lot number)
4- Life Technologies - goat anti-rabbit Alexa 647: A21245 (catalogue number) - 1623067 (lot number)

Validation

We performed validation of the primary antibody using anti-TH to label medullary C1 neurons which we published in previous studies (see PMID: 35722749). We performed controls to show an absence of non- specific staining from secondary antibodies; these data are depicted in the supplement file (fig s12).

Animals and other research organisms

Policy information about studies involving animals; ARRIVE guidelines recommended for reporting animal research, and Sex and Gender in Research

Laboratory animals

Wistar rats, male, adult (7-8 weeks old) and juvenile (4 weeks old), supplied by the Animal Facility of the Ribeirão Preto Medical School, University of São Paulo, Ribeirão Preto, Brazil.

Wild animals

No wild animals were used in this study.

Reporting on sex

Males were used as the hypothesis of this study was based on data gleaned from previous studies in male rats. Whilst we plan to perform a second study on female rats in the future, we do not have the financial resource to complete this for now. The present study has taken 3 years to complete and its duration exaggerated by global pandemic. We have indicated male rats in the abstract of the manuscript as per journal instruction.

Field-collected samples

The study did not involve samples collected from the field.

Ethics oversight

The Institutional Ethics Committee in Animal Experimentation (CEUA) of the Ribeirao Preto Medical School, University of Sao Paulo approved the experimental protocol (Protocol number 033/2017).

Note that full information on the approval of the study protocol must also be provided in the manuscript.

Flow Cytometry

Plots

Confirm that:

☒ The axis labels state the marker and fluorochrome used (e.g. CD4-FITC).

☐ The axis scales are clearly visible. Include numbers along axes only for bottom left plot of group (a 'group' is an analysis of identical markers).

☐ All plots are contour plots with outliers or pseudocolor plots.

☒ A numerical value for number of cells or percentage (with statistics) is provided.

Methodology

Sample preparation

Blood samples

Instrument

Becton Dickinson FACScan flow cytometer (Becton Dickinson Immunocytometry Systems, San Jose, CA, USA).

Software

DIVA-BD software (Becton Dickinson Immunocytometry Systems, San Jose, CA, USA).

Cell population abundance

Red blood cells were lysed using ACK (Ammonium-Chloride-Potassium) buffer and cells centrifuged at 410 g for 15 mins. Cell

pellets were resuspended in RPMI 1640 medium and viable cells counted using Trypan blue solution (0.4 %) on a Neubauer chamber. Briefly, cells were plated in 96-well microplates (2×106 cells/well) and blocked with anti-CD32 to prevent Fc-mediated non-specific binding. Cells were then incubated for 30 mins with color combinations of the monoclonal antibodies.

Gating strategy

For the analysis of the different cell populations, the following criteria was used: based on the parameters FSC-A (area) and FSC-H (height), initially single cells were selected, a process known as exclusion of doublets. Then, the parameters FSC-A (area) and SSC-A (granularity) were used to delimit the region where cells of small size and low complexity, such as B and T lymphocytes or delimit the region where cells of large size and high complexity, such as dendritic cells. Subsequently, B cells (CD45RA+ CD3-), CD4+T cells (CD3+CD4+), CD8+T cells (CD4- CD8+), NK (CD3-CD161+), the CD28 costimulatory molecule (CD4+CD28+; CD8+CD28+) and CD11a/LFA-1 in CD4 and CD8+T cells were characterized. The antibodies anti-CD45RA subset-phycoerythrin (PE), anti-CD3 allophycocyanin (APC, clone 1F4), anti-CD4- phycoerythrin-Cy7 (PE-Cy7; clone OX-35), anti-CD8-peridinin-chlorophyll-protein complex (PerCP, clone OX-8), anti-CD28-PE (phycoerythrin) and anti-CD11a- fluorescein isothiocyanate (FITC, clone WT.1) to identify adhesion molecules were used (BD Biosciences or eBiosciences San Diego, CA, USA).

☒ Tick this box to confirm that a figure exemplifying the gating strategy is provided in the Supplementary Information.
